# Cancer‐Associated Fibroblasts Promote Glucose Metabolic Reprogramming and Progression of Triple‐Negative Breast Cancer via Exosomal circFAD104‐Mediated Intercellular Communication

**DOI:** 10.1002/advs.77504

**Published:** 2026-08-29

**Authors:** Lei Wang, Xiaoyan Li, Yiran Liang, Yifei Wang, Yuhan Jin, Jianing Wang, Dan Luo, Yuxia Yang, Jingze Yang, Yaming Li, Tong Chen, Dianwen Han, Zekun Wang, Wenjing Zhao, Bing Chen, Lijuan Wang, Qifeng Yang

**Affiliations:** ^1^ Department of Breast Surgery, General Surgery Qilu Hospital of Shandong University Jinan Shandong People's Republic of China; ^2^ Biological Resource Center Qilu Hospital of Shandong University Jinan Shandong People's Republic of China; ^3^ Research Institute of Breast Cancer Shandong University Jinan Shandong People's Republic of China

**Keywords:** cancer‐associated fibroblasts, circFAD104, exosome, glycolysis, triple‐negative breast cancer

## Abstract

Triple‐negative breast cancer (TNBC) is characterized by high metastatic tendency and poor prognosis, largely driven by dynamic crosstalk between cancer cells and the tumor microenvironment (TME). Cancer‐associated fibroblasts (CAFs) are a major component of the TME, yet their functional contributions to TNBC progression remain incompletely understood. In this study, we isolated exosomes from patient‐derived CAFs and normal fibroblasts (NFs), and profiled their circRNA content using RNA sequencing. circFAD104 was significantly enriched in CAFs and their derived exosomes, with elevated stromal expression confirmed by qRT‐PCR and in situ hybridization (ISH). PKH26 labeling demonstrated that CAF‐derived exosomes efficiently deliver circFAD104 to TNBC cells, where it enhances cancer stemness, metastasis, and glycolysis. Mechanistically, circFAD104 acts as a molecular scaffold to promote the interaction between the E3 ubiquitin ligase MARCHF8 and its substrate PGM1, facilitating MARCHF8‐mediated K48‐linked ubiquitination and subsequent proteasomal degradation of PGM1. Loss of PGM1 redirects glucose flux from glycogen synthesis toward glycolysis, thereby fueling tumor progression. Clinically, high circFAD104 levels were correlated with poorer overall survival in breast cancer patients. These findings uncover a CAF‐exosomal circFAD104 axis that reprograms glucose metabolism to drive TNBC progression via the circFAD104/MARCHF8/PGM1 pathway, highlighting its potential as a stroma‐targeted biomarker and prognostic predictor.

## Graphical abstract

CAF‐derived exosomes deliver circFAD104 into TNBC cells, where it acts as a molecular scaffold that bridges the E3 ligase MARCHF8 and PGM1, promoting MARCHF8‐mediated K48‐linked ubiquitination and proteasomal degradation of PGM1. Loss of PGM1 redirects glucose‐phosphate flux from glycogen synthesis toward glycolysis, thereby driving stemness, EMT, and metastasis in TNBC.

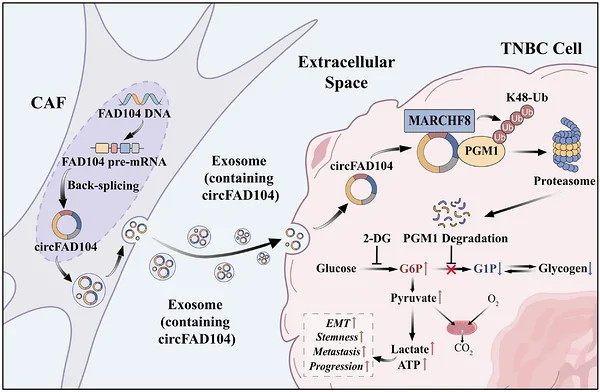

## 1 Introduction

Breast cancer remains the most frequently diagnosed malignancy and a leading cause of cancer‐related mortality among women worldwide [1]. Among its molecular subtypes, triple‐negative breast cancer (TNBC), which lacks estrogen receptors (ER), progesterone receptors (PR), and human epidermal growth factor receptor 2 (HER2), exhibits the most aggressive clinical behavior. Characterized by pronounced molecular heterogeneity, high rates of recurrence and metastasis, and limited therapeutic options, TNBC carries the poorest prognosis [2, 3]. Although it accounts for only 15–20% of all breast cancer cases, TNBC is responsible for approximately 25% of breast cancer‐related deaths [4], underscoring the urgent need to understand the molecular mechanisms underlying its aggressive phenotype.

Recent advances have shifted the focus from tumor cells alone to the broader tumor microenvironment (TME), recognizing that cancer progression arises from dynamic and multifaceted interplay between malignant cells and their stromal niche [5, 6, 7]. The TME comprises a complex ecosystem of immune cells, extracellular matrix (ECM), vasculature, and stromal fibroblasts, among which cancer‐associated fibroblasts (CAFs) constitute the most abundant stromal population [8]. Unlike normal fibroblasts (NFs), CAFs are educated and reprogrammed by tumor cells and acquire pro‐tumorigenic functions [9, 10]. These include ECM remodeling, secretion of immunosuppressive cytokines (e.g., IL‐6, Galectin‐1), and release of extracellular vesicles (EVs) that mediate intercellular communication. Emerging evidence reveals CAFs are functionally and metabolically heterogeneous rather than a uniform population [11, 12]. Despite these advances, the exosome‐mediated mechanisms by which CAFs communicate with TNBC cells and reshape tumor metabolism remain incompletely understood.

Extracellular vesicles, particularly exosomes (30–150 nm vesicles originating from multivesicular bodies), serve as key mediators of intercellular communication within the TME [13, 14]. Exosomes carry a variety of cargoes, including proteins, lipids, and diverse RNA species such as microRNAs (miRNAs), long non‐coding RNAs (lncRNAs), and circular RNAs (circRNAs), which can be stably transferred to recipient cells to reprogram their phenotype [14, 15]. For instance, CAF‐derived exosomal non‐coding RNAs have been shown to regulate therapy resistance, tumor cell survival, and metabolic adaptation in multiple cancer types.  [16, 17] CircRNAs, generated via back‐splicing of pre‐mRNA, form covalently closed loops that confer exceptional stability and resistance to RNase R degradation [18], making them well‐suited vehicles for sustained intercellular signaling. Indeed, CAF‐secreted exosomal circRNAs, such as circEIF3K and circIFNGR2, have been shown to promote tumor progression in colorectal and ovarian cancers  [19, 20]. Emerging evidence also implicates CAF‐derived exosomal circRNAs in breast cancer proliferation and metastasis [21, 22, 23]. Metabolic reprogramming, particularly the shift toward aerobic glycolysis, is a hallmark of cancer that supports rapid proliferation, stemness, and metastasis under nutrient‐limited TME conditions [24, 25]. Enhanced glycolysis is associated with poor prognosis in breast cancer and other malignancies [26, 27, 28]. Against this backdrop, stromal exosomal circRNAs may constitute an important but underexplored layer of metabolic regulation. Nevertheless, it remains unclear whether CAF‐derived exosomal circRNAs directly reshape glucose metabolism in TNBC cells, and how such metabolic rewiring contributes to cancer stemness and metastasis.

In this study, we identify circFAD104 as a CAF‐secreted exosomal cargo that links stromal communication to glucose metabolic reprogramming and TNBC progression. CAF‐derived exosomes deliver circFAD104 to TNBC cells, where it acts as a molecular scaffold to facilitate MARCHF8‐mediated K48‐linked ubiquitination and proteasomal degradation of phosphoglucomutase 1 (PGM1). This degradation redirects glucose flux from glycogen synthesis toward glycolysis, thereby enhancing cancer stemness, migration, and invasion. Clinically, elevated circFAD104 levels correlate with poor survival in TNBC patients. Collectively, our findings uncover a CAF‐exosomal circFAD104 axis that drives TNBC progression, highlighting circFAD104 as a stroma‐derived prognostic biomarker and a potential therapeutic vulnerability.

## 2 Materials and Methods

### 2.1 Clinical Specimens

Breast cancer tissues and paired adjacent normal tissues were collected from patients undergoing surgical resection at Qilu Hospital of Shandong University. Written informed consent was obtained from all participants, and the study was approved by the Ethics Committee of Qilu Hospital of Shandong University. For clinical correlation and survival analyses, TNBC patients were assigned to a discovery cohort and an independent validation cohort. Overall survival (OS) was defined as the interval from surgery to death or last follow‐up, and disease‐free survival (DFS) as the interval from surgery to recurrence, metastasis, death, or last follow‐up. Clinicopathological characteristics are summarized in Tables S1 and S2.

### 2.2 Isolation and Identification of Fibroblasts

Patient‐derived CAFs and NFs were isolated from pathologically confirmed TNBC tissues and paired adjacent non‐tumor tissues, respectively. Tissue fragments were minced into approximately 0.5 mm × 0.5 mm × 0.5 mm pieces and digested in DMEM supplemented with 10% FBS, 1 mg/mL collagenase I, and 0.1 mg/mL hyaluronidase at 37°C for 2 h with gentle agitation. After centrifugation, cells were resuspended in DMEM containing 10% FBS, seeded into 60‐mm dishes, and cultured at 37°C in 5% CO_2_. Adherent fibroblasts were purified by repeated PBS washing, and fibroblast identity was confirmed through immunofluorescence and Western blot analyses targeting specific markers.

### 2.3 Exosome Isolation, Identification, and Uptake Assay

CAFs and NFs were cultured in medium supplemented with exosome‐depleted FBS before conditioned medium collection. Exosomes were isolated from conditioned medium by differential ultracentrifugation. Briefly, conditioned medium was sequentially centrifuged at 300 × g for 10 min, 2000 × g for 10 min, and 10 000 × g for 70 min at 4°C to remove cells and debris, followed by ultracentrifugation at 100 000 × g for 70 min at 4°C. Exosome pellets were washed with 1× PBS, resuspended in DMEM or 1× PBS, filtered through 0.22‐µm filters, and quantified using a BCA Protein Assay Kit. For in vitro assays, 2 µg of exosomes were added to 1 × 10^5^ recipient TNBC cells. Exosome morphology and size distribution were assessed by transmission electron microscopy and nanoparticle tracking analysis, respectively. Exosome identity was confirmed by Western blotting for positive markers CD9 and CD63 and negative markers Calnexin and GM130. For uptake assays, exosomes were labeled with PKH26 dye according to the manufacturer's protocol and incubated with TNBC cells for 12 h, and internalization was observed via fluorescence microscopy (Olympus, Japan).

### 2.4 Cytosolic/Nuclear RNA Fractionation

Cytosolic and nuclear RNA fractions were isolated using the PARIS Kit (Invitrogen, USA) according to the manufacturer's instructions. GAPDH and U6 were used as cytosolic and nuclear fractionation controls, respectively. CircFAD104 abundance in each fraction was determined by qRT‐PCR.

### 2.5 Immunofluorescence (IF)

Cells were washed with 1× PBS, fixed with 4% paraformaldehyde for 20 min, permeabilized with 0.2% Triton X‐100 for 15 min, and blocked with 10% goat serum for 1 h at room temperature. They were then incubated with primary antibodies listed in Table S3 overnight at 4°C, followed by fluorescent secondary antibodies for 1 h at room temperature in the dark. Nuclei were counterstained with DAPI (Invitrogen, USA), and images were acquired using a fluorescence microscope (Olympus, Japan).

### 2.6 Fluorescence In Situ Hybridization (FISH)

FISH was performed using the GenePharma FISH kit (GenePharma, Shanghai, China). Cells were fixed with 4% paraformaldehyde for 20 min, permeabilized with 0.2% Triton X‐100 for 15 min, and hybridized with a Cy3‐labeled probe overnight at 37°C in the dark. After washing with pre‐warmed wash buffer, nuclei were counterstained with DAPI. Images were captured using a fluorescence microscope, and fluorescence intensity was quantified using ImageJ (RRID: SCR_003070).

### 2.7 In Situ Hybridization (ISH)

ISH was performed on 4‐µm TNBC tissue sections using the Enhanced Sensitive ISH Detection Kit I (BOSTER, China) according to the manufacturer's instructions. Briefly, sections were fixed with 4% paraformaldehyde, treated to block endogenous peroxidase activity, digested with pepsin, prehybridized, and incubated overnight at 37°C with a digoxin‐labeled probe. Signals were visualized using DAB (ZSGB‐BIO, China), and nuclei were counterstained with hematoxylin (Solarbio, China). CircFAD104 expression was evaluated separately in tumor‐cell and CAF‐enriched stromal compartments. The ISH score was calculated by multiplying the staining intensity score by the positive area score. Staining intensity was scored as 0, negative; 1, weak; 2, moderate; and 3, strong. The positive area was scored as 0, <5%; 1, 5–25%; 2, 26–50%; 3, 51–75%; and 4, 76–100%. Patients were stratified into circFAD104‐high and circFAD104‐low groups according to the ROC‐derived cutoff.

### 2.8 RNA Pull‐Down Assay and Mass Spectrometry

Biotin‐labeled circFAD104 sense, antisense, and truncated probes were synthesized by GenePharma (Shanghai, China). TNBC cells were lysed in RNA pull‐down lysis buffer supplemented with protease inhibitors and RNase inhibitor. Cell lysates were pre‐cleared with streptavidin magnetic beads and incubated with the indicated biotin‐labeled probes at 4°C with gentle rotation. Probe‐associated RNA‐protein complexes were captured using streptavidin magnetic beads and washed extensively with cold wash buffer. Bound proteins were eluted and analyzed by Western blotting. For mass spectrometry, proteins pulled down by circFAD104 sense or antisense control probes were separated by SDS‐PAGE and visualized by silver staining or Coomassie brilliant blue staining. Enriched protein bands or fractions were excised and subjected to LC‐MS/MS analysis by Beijing Qinglian Biotech Co., Ltd. Candidate circFAD104‐interacting proteins were prioritized based on enrichment over the antisense control and biological relevance to glucose metabolism.

### 2.9 RNA Immunoprecipitation (RIP) Assay

RIP assays were performed using the Magnetic RIP RNA‐Binding Protein Immunoprecipitation Kit (Millipore, USA) according to the manufacturer's instructions. Cells were lysed in RIP lysis buffer supplemented with RNase inhibitor and protease inhibitor cocktail. Cell lysates were incubated overnight at 4°C with magnetic beads conjugated with the indicated antibodies or species‐matched normal IgG. After washing, RNA‐protein complexes were digested with proteinase K, and bound RNAs were purified, reverse‐transcribed, and analyzed by qRT‐PCR using circFAD104‐specific primers. Relative enrichment was normalized to IgG control.

### 2.10 Co‐Immunoprecipitation (Co‐IP) Assay

Cells were lysed in IP‐compatible lysis buffer (Beyotime, China) supplemented with protease inhibitor cocktail. The lysates were incubated with the indicated primary antibodies or species‐matched normal IgG for 4 h at 4°C. Protein A/G agarose beads were then added and incubated overnight at 4°C. After washes with cold IP wash buffer, bound proteins were eluted with 2× SDS loading buffer by heating at 95°C for 10 min and analyzed by Western blotting.

### 2.11 Flow Cytometry (FCM)

Flow cytometry was performed to assess apoptosis, cell cycle distribution, cancer stem‐like cell populations, and CAF proportions in mouse tumor tissues. For apoptosis analysis, transfected TNBC cells were stained with PE Annexin V and 7‐AAD using the PE Annexin V Apoptosis Detection Kit I (BD Pharmingen) according to the manufacturer's instructions. For cell cycle analysis, cells were stained with cell cycle staining buffer (Multi Sciences, Hangzhou, China) in the dark. For cancer stem‐like cell detection, MDA‐MB‐468 cells were stained with PE‐CD44 and FITC‐CD24 antibodies (BD Biosciences) to identify CD44^+^CD24^−^ cells, whereas ALDH^+^ MDA‐MB‐231 cells were analyzed using the ALDEFLUOR Kit (STEMCELL Technologies). For tumor tissue flow cytometry, freshly harvested mouse tumors were dissociated into single‐cell suspensions. Cells were fixed, permeabilized, and stained for intracellular α‐SMA to evaluate α‐SMA^+^ CAF‐enriched populations. Data were acquired using a CytoFLEX flow cytometer (Beckman Coulter, Brea, CA, USA) and analyzed with CytExpert and FlowJo software.

### 2.12 ATP Level and Lactate Production Detection

ATP levels and lactate production were measured in transfected or exosome‐treated TNBC cells using the ATP Assay Kit (Nanjing Jiancheng Bioengineering Institute) and the Lactic Acid Assay Kit (Nanjing Jiancheng Bioengineering Institute) according to the manufacturers’ instructions. For 2‐deoxyglucose (2‐DG) rescue experiments, cells were treated with vehicle control or 10 mM 2‐DG for 24 h before metabolic measurements. Intracellular ATP levels were measured from cell lysates, whereas extracellular lactate levels were determined from culture supernatants collected after incubation in serum‐free medium.

### 2.13 Glycolysis Stress Test

The extracellular acidification rate (ECAR) was measured using the Seahorse XF Glycolysis Stress Test Kit (Agilent Technologies, CA, USA) on a Seahorse XFe96 Analyzer. TNBC cells were seeded into XF96 microplates and incubated overnight. Before analysis, cells were washed and equilibrated in Seahorse assay medium at 37°C without CO_2_. ECAR was measured after sequential injection of glucose, oligomycin, and 2‐DG according to the manufacturer's protocol. Glycolytic parameters, including basal glycolysis, glycolytic capacity, and glycolytic reserve, were calculated and normalized to cell number.

### 2.14 Patient‐Derived Organoid (PDO) Culture

Fresh TNBC tissues were minced and digested for 2 h at 37°C on an orbital shaker in DMEM/F12‐based digestion medium supplemented with 1% BSA, ITS‐G (BasalMedia, Shanghai, China), 5 µM Y‐27632 (MCE, NJ, USA), Primocin (InvivoGen, CA, USA), 10 mM HEPES (Thermo Fisher Scientific, MA, USA), 1000 U/mL hyaluronidase (Sigma‐Aldrich, MO, USA), and collagenase I/III (Worthington Biochemical Corporation, NJ, USA). The digested mixture was filtered through a 100‐µm cell strainer and centrifuged at 300 × g for 5 min at 4°C. The cell pellet was resuspended in digestion termination solution containing DMEM/F12, 0.1% BSA, and Primocin. After erythrocyte lysis with TAC buffer, the remaining cells were washed and resuspended in growth factor‐reduced Matrigel (R&D Systems, MN, USA) diluted 1:3 with organoid medium. The organoid medium consisted of DMEM/F12 supplemented with 0.5 µg/mL R‐spondin‐1 (BioLegend, CA, USA), 0.5 µg/mL neuregulin 1, 5 ng/mL FGF7, 5 ng/mL EGF, 0.1 µg/mL Noggin, 20 ng/mL FGF10 (all from PeproTech, NJ, USA), 500 nM A83‐01 (Tocris Bioscience, Bristol, UK), 5 µM Y‐27632 (MCE), 500 nM SB202190, 1.25 mM N‐acetyl‐L‐cysteine, 5 mM nicotinamide (all from Sigma‐Aldrich), 1× Primocin, 1× GlutaMAX, 1× HEPES, and 1× B27 (Gibco, Thermo Fisher Scientific, MA, USA). The organoid suspension was seeded into 48‐well plates and incubated at 37°C for 1 h to allow Matrigel solidification, followed by addition of 300 µL organoid medium. Fresh medium was replaced every 3 days, and organoids were passaged using TrypLE (Gibco). For functional assays, organoids were infected with circFAD104‐overexpressing or control lentivirus 48 h after seeding. After 48 h of infection, organoids were treated with vehicle or 10 mM 2‑DG, and the medium was replaced every 3 days with freshly prepared 2‑DG‑containing organoid medium. After 7 days, organoid growth was recorded using a light microscope with a 20× objective (ZEISS, Oberkochen, Germany), and organoid size was quantified from bright‐field images using ImageJ software.

### 2.15 Animal Experiments

All animal experiments were approved by the Animal Care and Use Committee of Shandong University. Female BALB/c nude mice aged 4–6 weeks were used. For subcutaneous xenograft assays, 5 × 10^6^ MDA‐MB‐231 cells stably expressing circFAD104, PGM1, or the corresponding control vectors were injected subcutaneously into the flanks of nude mice. For CAF co‐injection assays, 5 × 10^6^ MDA‐MB‐231 cells were injected alone or mixed with the stably transfected CAFs at a 1:1 ratio before subcutaneous implantation. Tumor length and width were measured every 5 days, and tumor volume was calculated as length × width^2^ / 2. After 3–4 weeks, mice were euthanized, and tumors were excised, photographed, weighed, and processed for HE staining, IHC, ISH or flow cytometry as indicated. For experimental lung metastasis assays, 1 × 10^6^ MDA‐MB‐231 cells stably overexpressing circFAD104, PGM1, or corresponding control vectors were injected via the tail vein. For bioluminescence monitoring, MDA‑MB‑231 cells were stably labeled with firefly luciferase. Mice were imaged on an IVIS Spectrum following intraperitoneal D‑luciferin injection, and lung photon flux was quantified using Living Image software. At the endpoint, all mice were sacrificed and lungs were harvested. Metastatic burden was evaluated by gross nodule counting and confirmed by HE staining of paraffin‐embedded lung sections.

### 2.16 Statistical Analysis

Statistical analyses were performed using GraphPad Prism 9.5.1 (GraphPad Software Inc., USA; RRID: SCR_002798) and R software version 4.4.2 (R Foundation, Vienna, Austria). Data are presented as mean ± standard deviation (SD) unless otherwise indicated. All experiments were performed with at least three independent biological replicates. Differences between two groups were analyzed using the two‐tailed Student's t‐test. Differences among multiple groups were analyzed using one‐way ANOVA followed by Tukey's post‐hoc test for multiple comparisons. The association between circFAD104 expression and clinico‐pathological variables was analyzed using the chi‐square test or Fisher's exact test, as appropriate. Correlations between variables were assessed using Pearson's correlation analysis. ROC curve analysis was performed to determine the optimal cutoff value of circFAD104 expression based on the clinical outcomes. Survival curves were generated using the Kaplan‐Meier method and compared using the log‐rank test. Hazard ratios (HRs) and 95% confidence intervals (CIs) were estimated using Cox regression analysis. *p* value < 0.05 was considered statistically significant.

## 3 Results

### 3.1 Identification of circFAD104 as a CAF‐Enriched and Exosome‐Delivered circRNA in TNBC

CAFs and normal fibroblasts (NFs) were isolated from breast cancer tissues and corresponding normal tissues of TNBC patients, respectively. Phenotypic validation confirmed the fibroblast lineage of CAFs and their paired NFs, as evidenced by spindle‐shaped morphology and strong expression of the pan‐fibroblast marker Vimentin, with no significant difference between groups (Figure S1A,B). Notably, CAFs displayed elevated levels of CAF activation markers, including α‐smooth muscle actin (α‐SMA), fibroblast activation protein (FAP), and fibroblast‐specific protein 1 (FSP‐1), as determined by IF and Western blot analyses (Figure S1A,B), consistent with an activated phenotype. We next isolated extracellular vesicles from the conditioned medium (CM) of NFs and CAFs using differential ultracentrifugation. Western blot analysis revealed that both NF‐derived exosomes (NF‐EXO) and CAF‐derived exosomes (CAF‐EXO) showed strong expression of exosomal markers CD9 and CD63, with absence of the endoplasmic reticulum marker Calnexin and the Golgi marker GM130 (Figure S2A), confirming their high purity. Transmission electron microscopy (TEM) revealed that both NF‐EXO and CAF‐EXO displayed the typical cup‐shaped and membrane‐enclosed vesicle structures (Figure S2B). Nanoparticle tracking analysis (NTA) confirmed a size distribution of 100–150 nm, consistent with the typical size of exosomes (Figure S2C). Importantly, PKH26‐labeled exosomes were efficiently internalized by TNBC cells, as demonstrated by strong cytoplasmic fluorescence after 12‐h co‐incubation (Figure S2D). These findings indicate that CAFs not only exhibit a highly activated phenotype but also secrete exosomes capable of being taken up by cancer cells, providing a basis for investigating exosome‐mediated molecular crosstalk in TNBC.

**FIGURE 1:**
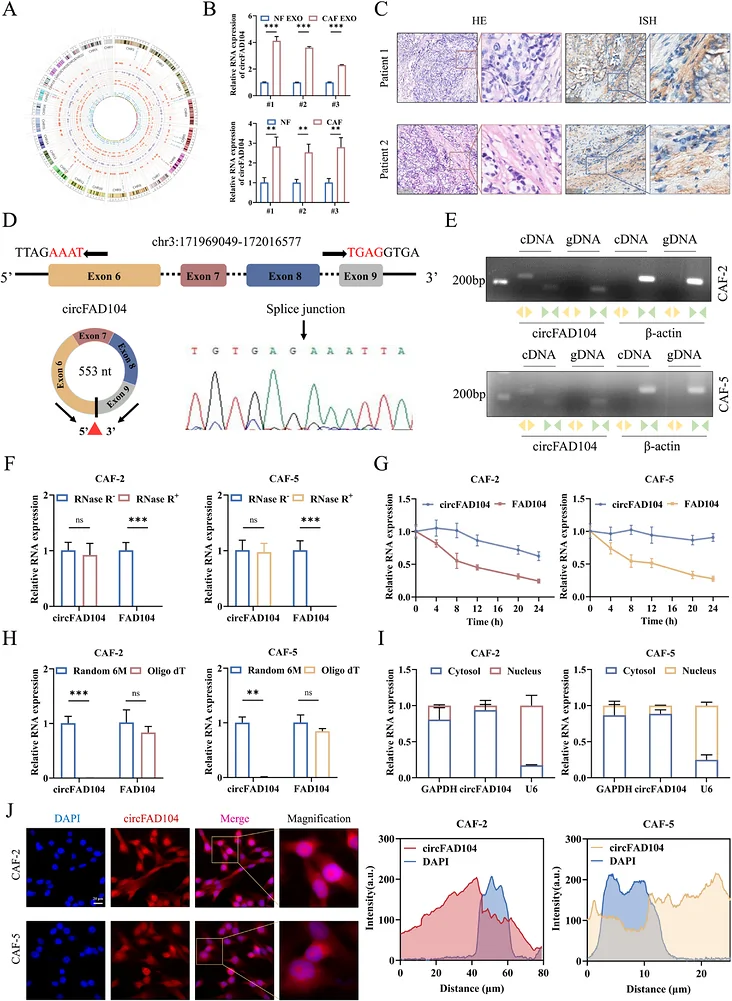
Identification of CAF‐enriched exosomal circFAD104 in TNBC. (A) Circos plot showing differentially expressed circRNAs in CAF‐ vs NF‐derived exosomes. (B) qRT‐PCR detection of circFAD104 expression in NFs, CAFs, and their corresponding exosomes from three patient‐matched pairs. (C) ISH staining of circFAD104 in TNBC tissue sections; HE staining showing tissue histology. Scale bars, 100 µm. (D) Schematic of circFAD104 genomic origin and back‐splicing pattern; Sanger sequencing of the head‐to‐tail junction. (E) PCR amplification of circFAD104 and β‐actin in CAF‐derived cDNA and gDNA using divergent and convergent primers. (F) qRT‐PCR analysis of circFAD104 and linear FAD104 mRNA in CAFs after RNase R treatment. (G) RNA half‐life assay of circFAD104 and linear FAD104 mRNA using actinomycin D. (H) qRT‐PCR amplification of circFAD104 and linear FAD104 with random hexamer or oligo (dT) primers. (I) qRT‐PCR quantification of circFAD104 in cytoplasmic and nuclear fractions of CAFs; GAPDH and U6 as fractionation controls. (J) FISH assay for subcellular localization of circFAD104 in CAFs; nuclei stained with DAPI. Scale bars, 20 µm. ns, not significant; **p < 0.01; ***p < 0.001.

Subsequently, we focused on characterizing the exosomal cargo derived specifically from these activated stromal cells. RNA sequencing was performed to compare the circRNA profiles of exosomes isolated from CAFs versus their paired NFs (Figure 1A). Given that exosomal circRNAs enriched in CAFs are likely to play pro‐tumorigenic roles and serve as potential biomarkers, we prioritized candidates with high fold changes and circBase annotations for validation. qRT‐PCR confirmed that circFAD104 (hsa_circ_0067990) was consistently enriched in CAF‐derived exosomes across three patient‐matched pairs (Figure 1B). CircFAD104 expression was also markedly higher in CAFs themselves compared to paired NFs (Figure 1B), suggesting active synthesis within this fibroblast population. Therefore, circFAD104 was chosen for further functional and mechanistic studies. In situ hybridization (ISH) analysis revealed that circFAD104 was detectable in both tumor epithelium and stroma, with markedly enriched expression in the tumor stroma (Figure 1C). Complementary qRT‐PCR assays on an expanded panel further validated that circFAD104 levels were substantially higher in CAFs than in TNBC cells and other breast cancer cell lines, with the highest expression observed in CAF‐2 and CAF‐5 (Figure S2E). These two CAF lines were therefore selected as models for subsequent functional and mechanistic studies to investigate the role of CAF‐derived circFAD104 in TNBC progression.

CircFAD104 is a 553‐nt circRNA derived from back‐splicing of exons 6–9 of the FAD104 gene on chromosome 3. To validate the circular nature of circFAD104, Sanger sequencing of the junction site was performed, confirming the predicted head‐to‐tail splicing (Figure 1D). Using divergent and convergent primers, we demonstrated that divergent primers amplified circFAD104 only in cDNA, not in gDNA, whereas convergent primers amplified both circFAD104 and β‐actin in cDNA and gDNA (Figure 1E). Treatment with RNase R resulted in near‐complete degradation of linear FAD104 mRNA, while circFAD104 remained largely intact (Figure 1F). Similarly, actinomycin D chase assays revealed that circFAD104 was much more stable compared to linear FAD104 mRNA (Figure 1G), confirming its enhanced stability. Additionally, circFAD104 was amplified only with random hexamer primers, not oligo (dT), indicating the absence of a poly (A) tail (Figure 1H). Subcellular fractionation and FISH assays showed that circFAD104 is predominantly localized in the cytoplasm of CAFs (Figure 1I,J). Collectively, these findings identify circFAD104 as a stable, CAF‐enriched, and exosome‐packaged circRNA, supporting its potential role in CAF‐TNBC cell communication.

### 3.2 CAF‐Derived Exosomal circFAD104 Promotes Stemness, Metastasis, and Glycolytic Reprogramming in TNBC Cells

To determine whether circFAD104 is functionally required for the pro‐tumorigenic effects of CAFs, we designed a small interfering RNA (siRNA) specifically targeting the back‐splicing junction of circFAD104 (Figure S3A). qRT‐PCR confirmed efficient knockdown of circFAD104 in CAFs without affecting expression of its linear host transcript, FAD104 (Figure S3B). We next evaluated the functional consequences of circFAD104 knockdown by treating TNBC cells with CM from CAFs transfected with si‐NC or si‐circFAD104. CM from control CAFs (si‐NC) significantly enhanced proliferation and migration of MDA‐MB‐231 and MDA‐MB‐468 cells compared to CM from NFs or untreated controls, whereas CM from si‐circFAD104‐transfected CAFs failed to promote these malignant phenotypes (Figure S3C,D). These CM‐based assays suggested that CAF‐derived circFAD104 contributes to the pro‐tumorigenic activity of the CAF secretome.

**FIGURE 2:**
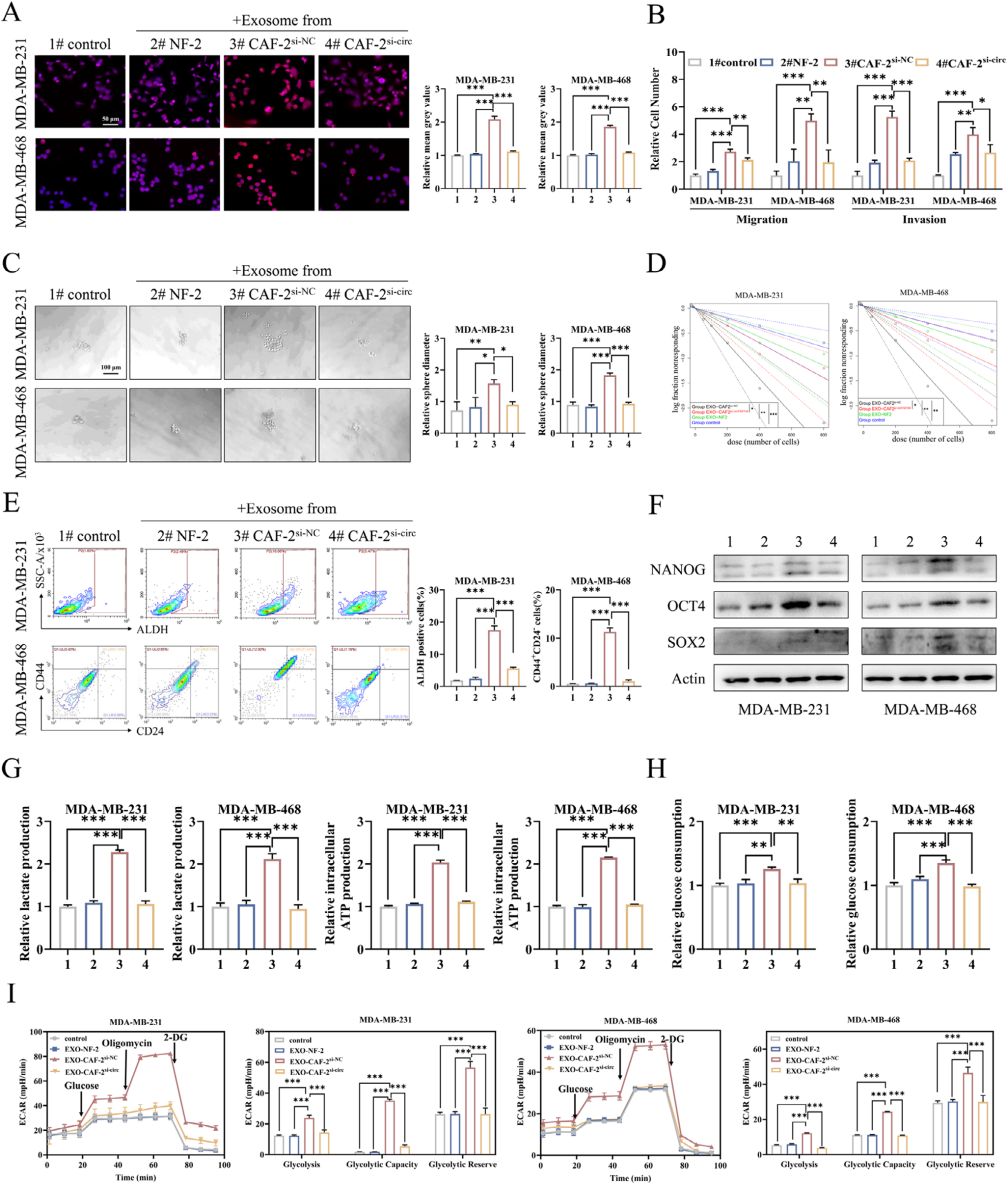
Exosomal circFAD104 regulates TNBC stemness, metastasis, and glycolysis. (A) FISH staining of TNBC cells treated with NF‐2 EXO, CAF‐2^si‐NC^ EXO, or CAF‐2^si‐circFAD104^ EXO. Scale bars, 50 µm. (B) Transwell assay of TNBC cells after exosome treatment. (C) Sphere formation assay of TNBC cells after exosome treatment. Scale bars, 100 µm. (D) Extreme limiting dilution analysis (ELDA) of TNBC cells after exosome treatment. (E) Flow cytometry analysis of ALDH^+^ cells (MDA‐MB‐231) and CD44^+^CD24^−^ cells (MDA‐MB‐468) after exosome treatment. (F) Western blot analysis of stemness‐related markers in TNBC cells after exosome treatment. (G) Detection of lactate and ATP production in TNBC cells after exosome treatment. (H) Measurement of glucose consumption levels in TNBC cells after exosome treatment. (I) Seahorse XF Glycolysis Stress Test for ECAR in TNBC cells after exosome treatment. Groups: 1, control; 2, NF‐2 EXO; 3, CAF‐2^si‐NC^ EXO; 4, CAF‐2^si‐circFAD104^ EXO. *p < 0.05; **p < 0.01; ***p < 0.001.

We further investigated whether circFAD104 is transferred from CAFs to TNBC cells via exosomes to exert its pro‐tumorigenic effects. TNBC cells were treated with exosomes from NFs, or from CAFs transfected with si‐NC or si‐circFAD104 (Figure S4A). qRT‐PCR analysis revealed that circFAD104 levels were significantly elevated in TNBC cells treated with CAF‐2^si‐NC^ exosomes, whereas this increase was attenuated in cells treated with CAF‐2^si‐circFAD104^ exosomes (Figure S4B). Notably, no changes were observed in linear FAD104 mRNA levels across groups (Figure S4C), indicating that the elevated circFAD104 in recipient cells is derived from exosomal transfer rather than transcriptional upregulation of the host gene. FISH further demonstrated strong cytoplasmic accumulation of circFAD104 in TNBC cells treated with CAF‐2^si‐NC^ exosomes, while signals were weak in other groups (Figure 2A). Functionally, CAF‐2^si‐NC^ exosomes enhanced TNBC cell proliferation, migration, and invasion, whereas these effects were attenuated by circFAD104 knockdown in CAFs (Figure 2B, Figure S4D–G). We then examined the morphological and molecular hallmarks of EMT. Phalloidin staining revealed that CAF‐2^si‐NC^ exosome‐treated cells adopted a spindle‐shaped, mesenchymal morphology, whereas cells in other groups exhibited an epithelial cobblestone‐like appearance (Figure S4H). At the molecular level, CAF‐2^si‐NC^ exosomes upregulated mesenchymal markers (Fibronectin, N‐cadherin, Vimentin) and downregulated the epithelial marker E‐cadherin (Figure S4I). These EMT changes were abrogated when circFAD104 was knocked down in CAFs. Moreover, TNBC cells treated with CAF‐2^si‐NC^ exosomes formed significantly larger spheres (Figure 2C). Extreme limiting dilution analysis (ELDA) revealed a higher frequency of cancer stem‐like cells (CSCs) compared to other groups (Figure 2D). Flow cytometry further confirmed an increased proportion of ALDH^+^ or CD44^+^CD24^−^ populations in the CAF‐2^si‐NC^ exosome group, which were reversed upon circFAD104 knockdown (Figure 2E). Consistently, western blot analysis showed that CAF‐2^si‐NC^ exosome increased the expression of stemness‐related markers (Figure 2F). Given that metabolic reprogramming is a key mechanism supporting cancer stemness, we further evaluated the effect of CAF‐derived exosomal circFAD104 on glycolytic metabolism in TNBC cells. Treatment with CAF‐2^si‐NC^ exosomes significantly increased lactate production, ATP levels, and glucose consumption (Figure 2G,H), whereas exosomes from CAF‐2^si‐circFAD104^ failed to induce these metabolic changes. Consistently, Seahorse XFe96 analysis showed that CAF‐2^si‐NC^ exosomes markedly enhanced basal glycolysis, glycolytic capacity, and glycolytic reserve. In contrast, CAF‐2^si‐circFAD104^ exosomes produced no such enhancement (Figure 2I), indicating that exosomal circFAD104 is required for CAF‐mediated glycolytic reprogramming. Results from CAF‐5 further validated the enhanced effects of exosomal circFAD104 on metastatic potential and glycolysis of TNBC cells (Figures S5 and S6). Collectively, these results indicate that CAFs deliver functional circFAD104 to TNBC cells through exosomes, and this transfer is critical for eliciting tumor‐promoting effects.

Having established that CAF‐derived exosomal circFAD104 is required for CAF‐mediated effects, we next examined whether circFAD104 itself is sufficient to drive malignant phenotypes in TNBC cells. We generated a circFAD104‐overexpressing construct using the pLCDH‐ciR vector (Figure S7A). qRT‐PCR confirmed the upregulation of circFAD104 in MDA‐MB‐231 and MDA‐MB‐468 cells without altering the expression of its linear host gene FAD104 (Figure S7B), validating the specificity of circRNA overexpression. Functional assays revealed that circFAD104 overexpression significantly enhanced the proliferative, migratory, invasive, and pro‐angiogenic abilities of TNBC cells (Figure S7C–E). Concomitantly, circFAD104‐overexpressing cells adopted a spindle‐shaped, mesenchymal morphology (Figure S7F), accompanied by E‐cadherin downregulation and upregulation of mesenchymal markers (Figure S7G), indicative of EMT induction. Flow cytometry analysis revealed that circFAD104 overexpression promoted cell cycle progression (Figure S8A), accompanied by decreased apoptosis (Figure S8B). Moreover, circFAD104 promoted cancer stemness, as evidenced by increased tumor sphere formation (Figure S8C), elevated frequencies of ALDH^+^ and CD44^+^CD24^−^ populations (Figure S8D), and upregulated stemness‐associated proteins (Figure S8E). Notably, circFAD104 also drove glucose metabolic reprogramming. Seahorse XFe96 analysis demonstrated that circFAD104‐overexpressing cells exhibited significantly higher ECAR (Figure S8F), which was supported by upregulated protein levels of key glycolytic enzymes (Figure S8G). Metabolite measurements revealed increased glucose consumption, lactate production, ATP synthesis, and glucose‐6‐phosphate (G6P) levels, alongside reduced glucose‐1‐phosphate (G1P), further confirming that circFAD104 enhances glycolytic flux in TNBC cells (Figure S8H–L). Loss‐of‐function experiments were performed using siRNA targeting the back‐splicing junction of circFAD104 in TNBC cells, and knockdown efficiency was confirmed by qRT‐PCR without affecting linear FAD104 (Figure S9A). Consistently, circFAD104 knockdown suppressed proliferation, migration, invasion, angiogenesis, and EMT (Figure S9B–E), while promoting G1 arrest and apoptosis (Figure S10A,B). Furthermore, circFAD104 knockdown attenuated stemness and glycolytic activity (Figure S10C–L). These results collectively demonstrate that circFAD104 is essential for the aggressive phenotype of TNBC cells.

### 3.3 Glycolytic Activation Mediates the Oncogenic Effects of circFAD104 in TNBC

**FIGURE 3:**
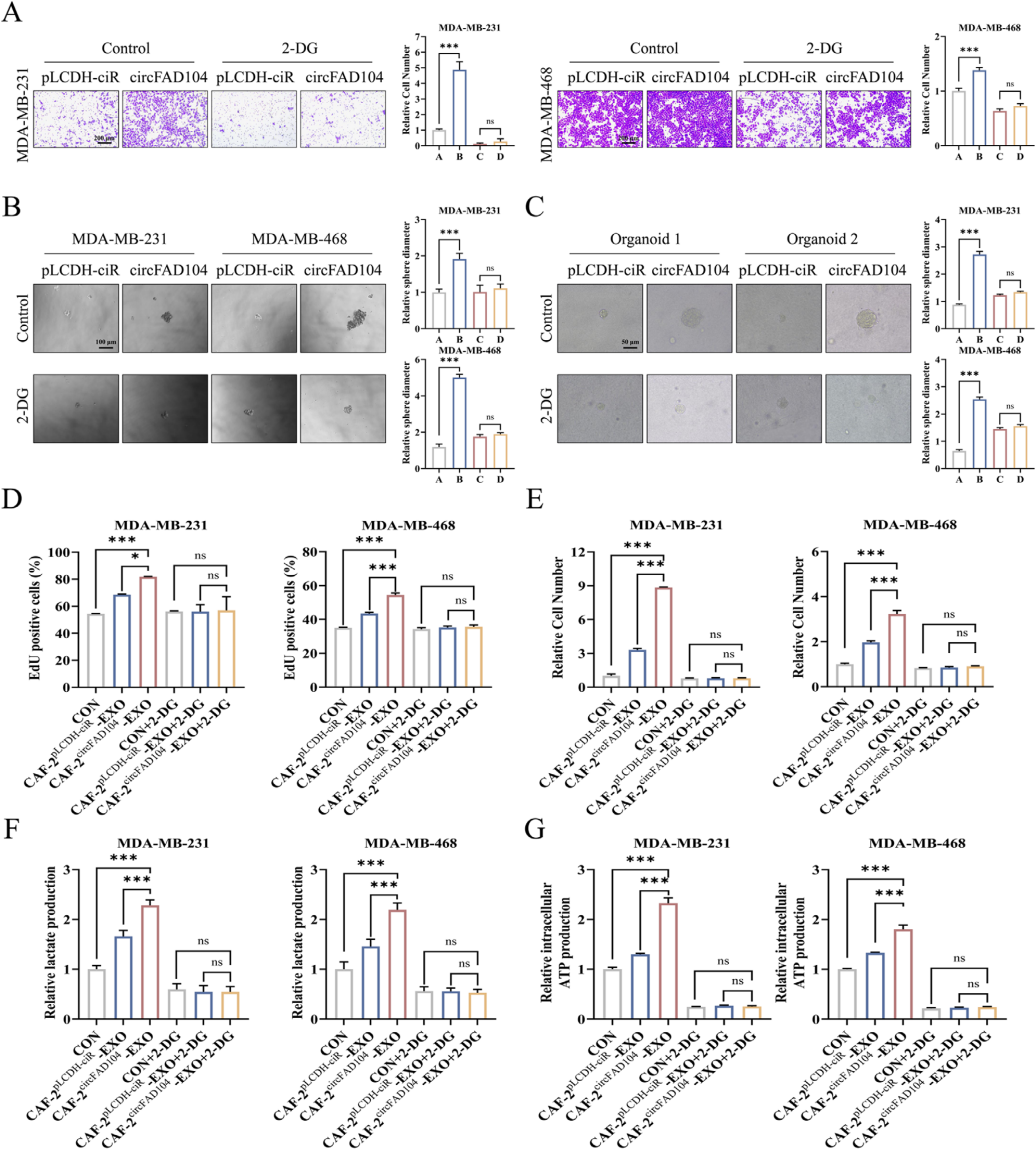
Exosomal circFAD104 regulates malignant phenotypes of TNBC by activating glycolytic metabolism. (A‐C) Transwell migration assay (Scale bars, 200 µm) (A), sphere formation assay (Scale bars, 100 µm) (B), and patient‐derived organoid (PDO) growth assays (Scale bars, 50 µm) (C) were performed to evaluate the effect of circFAD104 overexpression with or without 2‐DG treatment. (D‐G) 2‐DG rescue experiments in TNBC cells treated with circFAD104‐overexpressing CAF‐2‐derived exosomes: (D) EdU assay, (E) Transwell migration assay, (F) lactate production, and (G) ATP production. ns, not significant; ***p < 0.001.

To determine whether the oncogenic phenotypes driven by circFAD104 are functionally dependent on glycolytic activation, we performed pharmacological inhibition experiments in TNBC cells using 2‐deoxy‐D‐glucose (2‐DG), a glucose analog and glycolysis inhibitor. Importantly, 2‐DG did not alter circFAD104 expression (Figure S11A,B), ensuring that the subsequent phenotypic rescue reflected glycolytic inhibition rather than a change in circFAD104 abundance. Notably, 2‐DG significantly attenuated the circFAD104‐driven enhancement of core malignant behaviors, including cellular proliferation and migration (Figure 3A, Figure S11C–F). The pro‐stemness effect of circFAD104, evidenced by increased tumor sphere formation capacity, was also largely abrogated upon 2‐DG co‐treatment (Figure 3B). The physiological relevance of this metabolic dependency was further underscored by reproducing this rescue effect in patient‐derived organoid (PDO) models, where 2‐DG treatment also reversed the tumor‐promoting influence of circFAD104 overexpression (Figure 3C).

Next, we tested whether this glycolytic dependency was also observed in CAF‐tumor cell communication mediated by exosomal circFAD104. We modeled the delivery pathway by treating TNBC cells with exosomes isolated from circFAD104‐overexpressing CAFs. 2‐DG did not affect the exosome‐mediated delivery of circFAD104 to recipient TNBC cells (Figure S12A), indicating that its inhibitory effects were not due to impaired circFAD104 transfer. CAF‐derived circFAD104‐rich exosomes recapitulated the pro‐tumorigenic effects observed upon direct circFAD104 overexpression in cancer cells, whereas concurrent 2‐DG treatment markedly attenuated these exosome‐induced malignant phenotypes (Figure 3D–G, Figures S12B–E and S13). Collectively, these rescue experiments span both intrinsic (overexpression) and extrinsic (exosomal‐transfer) models. In both, the tumor‐promoting functions of circFAD104 were mediated, at least in part, by enhanced glycolysis, linking this exosomal circRNA to metabolic reprogramming in TNBC.

### 3.4 CircFAD104 Physically Interacts With PGM1 and Promotes its K48‐Linked Ubiquitination and Proteasomal Degradation

**FIGURE 4:**
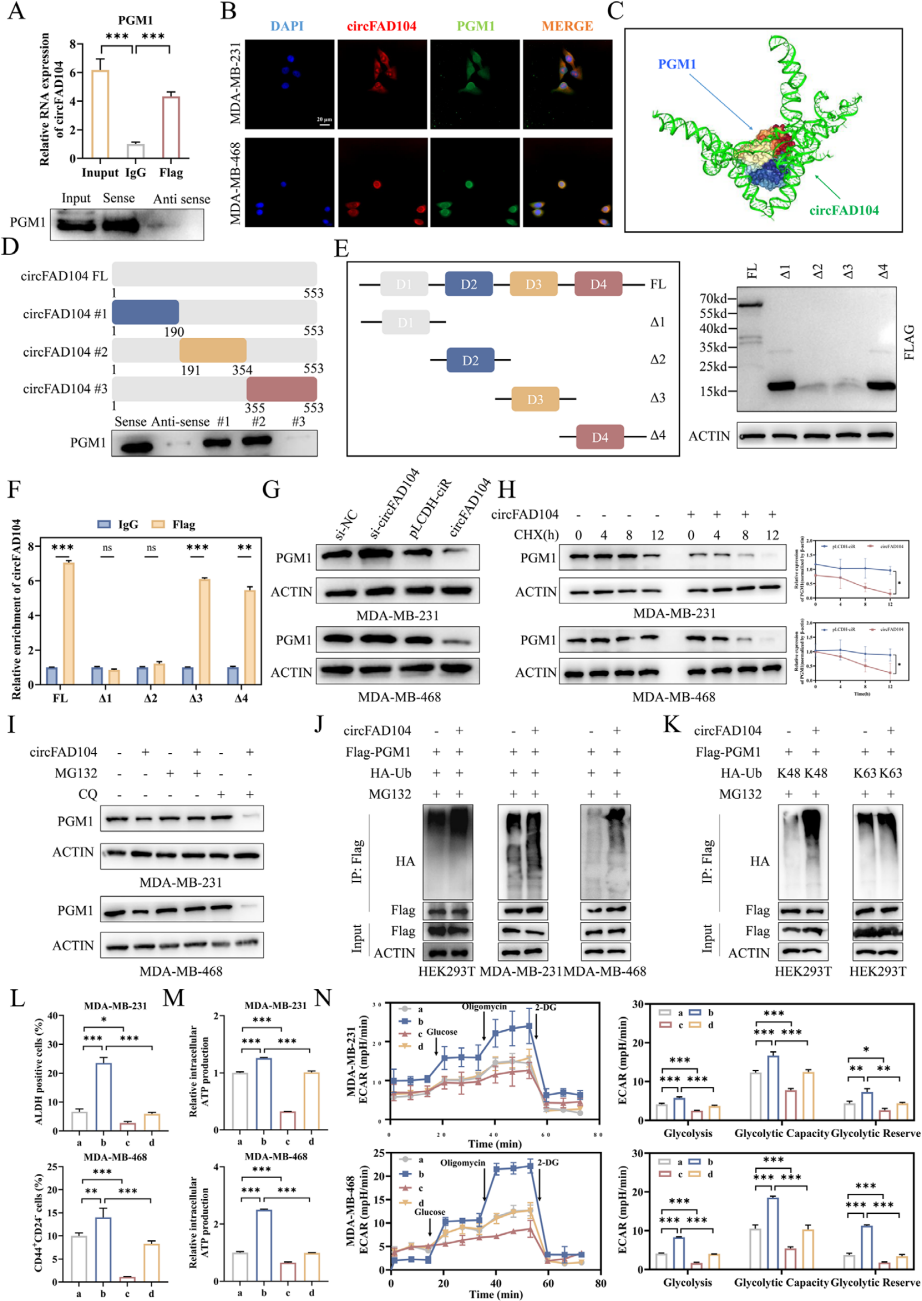
circFAD104 interacts with PGM1 and regulates its stability. (A) RIP and RNA pull‐down assays validating the interaction between circFAD104 and PGM1 in TNBC cells. (B) FISH‐IF analysis showing cytoplasmic colocalization of circFAD104 and PGM1 in TNBC cells; nuclei were stained with DAPI. Scale bars, 20 µm. (C) HDOCK‐predicted structural model of the circFAD104‐PGM1 interaction. (D) RNA pull‐down assay using truncated circFAD104 probes followed by Western blot detection of PGM1. (E) Western blot confirmation of FLAG‐tagged PGM1 domain fragments corresponding to D1‐D4. (F) RIP assay using an anti‐FLAG antibody to map circFAD104 binding to individual PGM1 domains. (G) Western blot analysis of PGM1 protein levels in TNBC cells with circFAD104 knockdown or overexpression. (H) Cycloheximide (CHX) chase assay assessing PGM1 protein stability after circFAD104 overexpression. (I) Western blot analysis of PGM1 levels in circFAD104‐overexpressing TNBC cells treated with MG132 or chloroquine (CQ). (J) Immunoprecipitation assay detecting total PGM1 ubiquitination after circFAD104 overexpression. (K) Ubiquitination assay detecting K48‐ and K63‐linked ubiquitination of PGM1 after circFAD104 overexpression. (L‐N) PGM1 rescue experiments in circFAD104‐overexpressing TNBC cells: flow cytometric analysis of cancer stem‐like cell populations (L), ATP production (M), and ECAR (N). Groups: a, control; b, circFAD104; c, PGM1; d, circFAD104 + PGM1. ns, not significant; *p < 0.05; **p < 0.01; ***p < 0.001.

To elucidate the molecular mechanism by which circFAD104 drives glycolytic reprogramming and malignant phenotypes in TNBC cells, we first sought to identify circFAD104‐interacting proteins. RNA pull‐down using a biotin‐labeled circFAD104 sense probe, followed by mass spectrometry (MS), identified 160 candidate proteins enriched by circFAD104 compared with the antisense control (Figure S14A). KEGG pathway enrichment analysis showed that several enriched proteins were involved in glycolysis/gluconeogenesis, including PDHB, PGM1, and GALM (Figure S14B). Pan‐cancer and breast cancer subtype analyses showed that, among these three candidates, PGM1 was consistently downregulated in tumor tissues, including TNBC, whereas PDHB and GALM showed more variable expression patterns across breast cancer subtypes (Figure S14C,D). Their expression patterns in breast cancer were further characterized at the bulk, single‐cell, and spatial transcriptomic levels (Figure S15). We then examined the functional roles of PDHB and GALM in TNBC cells, and their knockdown and overexpression efficiencies were confirmed by qRT‐PCR (Figure S16A,B). PDHB knockdown enhanced migration and lactate production, whereas its overexpression suppressed these phenotypes (Figure S16C–E), consistent with its known role in pyruvate oxidation. In contrast, GALM manipulation had no significant effects on migration, and its effects on ATP and lactate production were minimal and not consistent across cell lines or conditions (Figure S16F–H). Critically, RIP assays showed no significant enrichment of circFAD104 by either PDHB or GALM (Figure S16I,J), in contrast to the clear enrichment observed with PGM1 (Figure 4A). Therefore, we focused on PGM1, which was identified by MS as a high‐confidence circFAD104 interactor (Figure S17A). The interaction between circFAD104 and PGM1 was further validated by RNA pull‐down assay (Figure 4A). FISH‐IF analysis showed cytoplasmic colocalization of endogenous circFAD104 and PGM1 in TNBC cells (Figure 4B, Figure S17B), supporting their spatial proximity. Molecular docking using HDOCK predicted a plausible circFAD104‐PGM1 interaction interface (Figure 4C). Guided by secondary‐structure prediction of circFAD104 (Figure S17C), we designed truncated probes and performed RNA pull‐down to map the regions required for PGM1 binding. The 1–190 nt and 191–354 nt regions were necessary for efficient association with PGM1 (Figure 4D). In parallel, because PGM1 contains four conserved domains (D1‐D4) [29], we constructed FLAG‐tagged PGM1 domain fragments and confirmed their expected sizes and expression by Western blot (Figure 4E). RIP assays with an anti‐FLAG antibody showed that circFAD104 preferentially associated with the D3 and D4 domains (Figure 4F). Together, these results demonstrate that circFAD104 physically interacts with PGM1 in the cytoplasm through defined RNA regions and PGM1 domains.

We next examined the functional consequence of circFAD104‐PGM1 binding. Neither overexpression nor knockdown of circFAD104 altered PGM1 mRNA levels (Figure S18A), suggesting that circFAD104 regulates PGM1 post‐transcriptionally. In contrast, Western blot analysis showed that circFAD104 knockdown increased PGM1 protein levels, whereas circFAD104 overexpression reduced PGM1 abundance (Figure 4G). Immunofluorescence staining further confirmed a decreased cytoplasmic PGM1 signal in circFAD104‐overexpressing cells (Figure S18B). Cycloheximide chase assays showed that circFAD104 overexpression promoted PGM1 protein degradation, whereas circFAD104 knockdown attenuated this degradation (Figure 4H, Figure S18C), indicating that circFAD104 negatively regulates PGM1 protein stability.

To determine the degradation pathway involved, TNBC cells were treated with the proteasome inhibitor MG132 or the lysosome inhibitor chloroquine (CQ). MG132, but not CQ, rescued the circFAD104‐induced reduction in PGM1 (Figure 4I, Figure S18D,E), indicating that circFAD104 promotes proteasome‐dependent degradation of PGM1. Immunoprecipitation assays further showed that circFAD104 overexpression enhanced total PGM1 ubiquitination (Figure 4J). Linkage‐specific ubiquitination assays revealed that circFAD104 selectively increased K48‐linked polyubiquitination of PGM1, but not K63‐linked ubiquitination (Figure 4K). Since K48‐linked ubiquitination is a canonical signal for proteasomal degradation [30], these results indicate that circFAD104 promotes PGM1 degradation via the K48‐linked ubiquitination‐proteasome pathway.

### 3.5 PGM1 Suppresses TNBC Malignant Phenotypes by Restraining Glycolytic Metabolism

While PGM1 has been implicated in various cancers, such as hepatocellular carcinoma [31], lung cancer [32], and ovarian cancer [33], its role in breast cancer, and particularly TNBC, remains incompletely defined. Analysis of the TCGA‐BRCA cohort showed that PGM1 mRNA levels were significantly lower in breast cancer tissues than in adjacent normal tissues (Figure S19A). This downregulation was further validated in two independent datasets (GSE22820 and GSE62228) and at the protein level using CPTAC data (Figure S19B,C), supporting a potential tumor‐suppressive role for PGM1 in breast cancer. To define the cellular context of PGM1 expression, we analyzed multiple single‐cell RNA‐seq datasets from breast cancer and TNBC patients. PGM1 was detected mainly in malignant epithelial cells and fibroblasts (Figure S19D–F). Spatial transcriptomic analysis further revealed a heterogeneous PGM1 distribution in TNBC tissues, with detectable expression in tumor nests and lower expression at the tumor‐normal junction and in peripheral stromal regions (Figure S19G–J). Together, these data indicate that PGM1 is reduced at the bulk tumor level while showing cell‐type‐ and spatial‐context‐dependent distribution within breast cancer tissues.

We next investigated the functional role of PGM1 in TNBC cells. PGM1 knockdown and overexpression were confirmed at both the mRNA and protein levels (Figure S20A,B). Functionally, PGM1 knockdown enhanced proliferation, colony formation, and EdU incorporation, whereas PGM1 overexpression produced the opposite effects (Figure S20C–E). Consistently, PGM1 knockdown increased wound closure, migration, invasion, and sphere formation, while PGM1 overexpression suppressed these phenotypes (Figure S21A–C). To validate this tumor‐suppressive function in vivo, we established subcutaneous xenograft and experimental lung‐metastasis models using MDA‐MB‐231 cells stably overexpressing PGM1 or vector control. PGM1 overexpression significantly reduced tumor volume and weight in the subcutaneous model (Figure S21D) and markedly decreased the pulmonary metastatic burden, resulting in fewer gross lung metastatic nodules (Figure S21E). These results demonstrate that PGM1 suppresses TNBC malignant phenotypes both in vitro and in vivo.

Given the central role of PGM1 in glucose‐phosphate metabolism, we next examined whether it regulates glycolytic activity in TNBC cells. Pathway enrichment analysis of PGM1‐associated genes closely linked PGM1 to metabolic processes, particularly glucose metabolism (Figure S22A). GSVA in the TCGA‐BRCA cohort and two TNBC datasets (GSE11121 and GSE21653) showed that high PGM1 expression was associated with enrichment of galactose and related glucose‐phosphate metabolic pathways (Figure S22B). Because PGM1 catalyzes the interconversion of G1P and G6P, we then assessed glycolytic flux and glucose‐phosphate metabolites. PGM1 knockdown increased basal glycolysis, glycolytic capacity, and glycolytic reserve (Figure S23A). It also increased glucose consumption, lactate production, ATP, and G6P, while reducing G1P (Figure S23B–F). In contrast, PGM1 overexpression exerted the opposite effects (Figure S23). These findings indicate that PGM1 restrains glycolysis by controlling the G1P/G6P balance, and that its loss shifts glucose‐phosphate metabolism toward a pro‐glycolytic state in TNBC cells.

To determine whether PGM1 suppression mediates circFAD104‐driven phenotypes, we performed PGM1 re‐expression rescue experiments. Western blot confirmed that exogenous PGM1 restored PGM1 levels in circFAD104‐overexpressing TNBC cells (Figure S24A,B). Functionally, PGM1 re‐expression attenuated circFAD104‐induced proliferation, migration, and invasion (Figure S24C–G). It also reduced circFAD104‐induced increases in sphere formation and metabolite output, and partially restored the G1P/G6P balance (Figure S25), consistent with the stemness and metabolic rescue shown in Figure 4L–N. These results identify PGM1 as a key downstream effector of circFAD104‐mediated metabolic and malignant phenotypes. Since circFAD104 binds the D3 and D4 domains of PGM1, we asked whether disrupting this interaction could attenuate circFAD104‐driven phenotypes. We generated a truncated construct containing only the D3‐D4 region (PGM1‐Δ3+4) to serve as a competitive decoy (Figure S26A). Overexpression of PGM1‐Δ3+4 partially reduced circFAD104‐induced malignant behaviors (Figure S26B–F), consistent with the functional importance of the circFAD104‐PGM1 interaction. Collectively, these findings establish PGM1 as a tumor‐suppressive regulator of glucose metabolic fate in TNBC: circFAD104‐mediated destabilization of PGM1 redirects glucose‐phosphate metabolism toward glycolysis, thereby promoting TNBC malignant progression.

### 3.6 CircFAD104 Functions as a Molecular Scaffold to Facilitate MARCHF8‐Mediated K48‐Linked Ubiquitination and Proteasomal Degradation of PGM1

**FIGURE 5:**
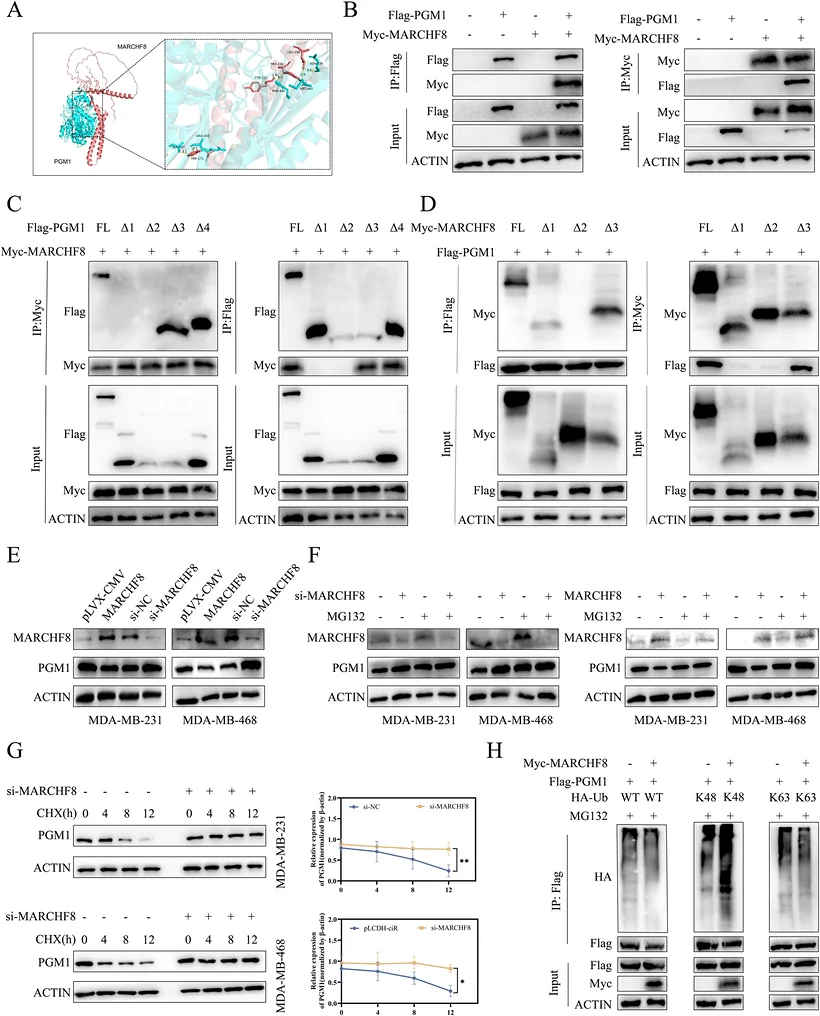
MARCHF8 acts as an E3 ligase and mediates PGM1 ubiquitination. (A) Schematic of the binding mode between E3 ubiquitin ligases and PGM1. (B) Exogenous co‐IP assays to confirm the interaction between MARCHF8 and PGM1. (C) Co‐IP assay with PGM1 truncation mutants to map MARCHF8 binding domains. (D) Co‐IP assay with MARCHF8 truncation mutants to map PGM1 binding domains. (E) Western blot analysis of PGM1 protein levels in TNBC cells with MARCHF8 overexpression or knockdown. (F) Western blot of PGM1 levels in TNBC cells with MARCHF8 manipulation and MG132 treatment. (G) CHX chase assay to assess PGM1 protein half‐life in TNBC cells with MARCHF8 manipulation. (H) Ubiquitination assay to detect K48‐linked polyubiquitination of PGM1 by MARCHF8. *p < 0.05; **p < 0.01.

To elucidate how circFAD104 promotes proteasomal degradation of PGM1, we sought to identify the E3 ubiquitin ligase responsible for PGM1 ubiquitination. By integrating UbiBrowser prediction, molecular docking, and molecular dynamics simulation, we prioritized MARCHF8 as a candidate E3 ligase for PGM1 (Figure 5A, Figure S27A). Exogenous and endogenous co‐IP assays confirmed the physical interaction between MARCHF8 and PGM1 (Figure 5B, Figure S27B). Immunofluorescence analysis further showed their colocalization (Figure S27C). MARCHF8 mainly contains an N‐terminal RING domain and two C‐terminal transmembrane regions (TMs) [34]. To delineate the MARCHF8 region required for PGM1 binding, we generated a series of Myc‐tagged MARCHF8 truncation mutants (Figure S27D). Co‐IP assays using these mutants showed that the RING‐TM1 domain of MARCHF8 was required for the interaction with the D3 and D4 domains of PGM1 (Figure 5C,D). We next examined whether MARCHF8 regulates PGM1 protein stability. MARCHF8 overexpression reduced PGM1 protein levels, whereas MARCHF8 knockdown elevated PGM1 abundance (Figure 5E, Figure S27E), indicating that MARCHF8 negatively regulates PGM1 expression. Treatment with the proteasome inhibitor MG132 rescued the MARCHF8‐induced reduction in PGM1 protein levels (Figure 5F), supporting proteasome‐dependent degradation. Consistently, cycloheximide chase assays showed that MARCHF8 knockdown enhanced the protein stability of PGM1 (Figure 5G). Ubiquitination assays further demonstrated that MARCHF8 selectively promoted K48‐linked, but not K63‐linked, polyubiquitination of PGM1 (Figure 5H). These results identify MARCHF8 as an E3 ligase that promotes K48‐linked ubiquitination and proteasomal degradation of PGM1.

**FIGURE 6:**
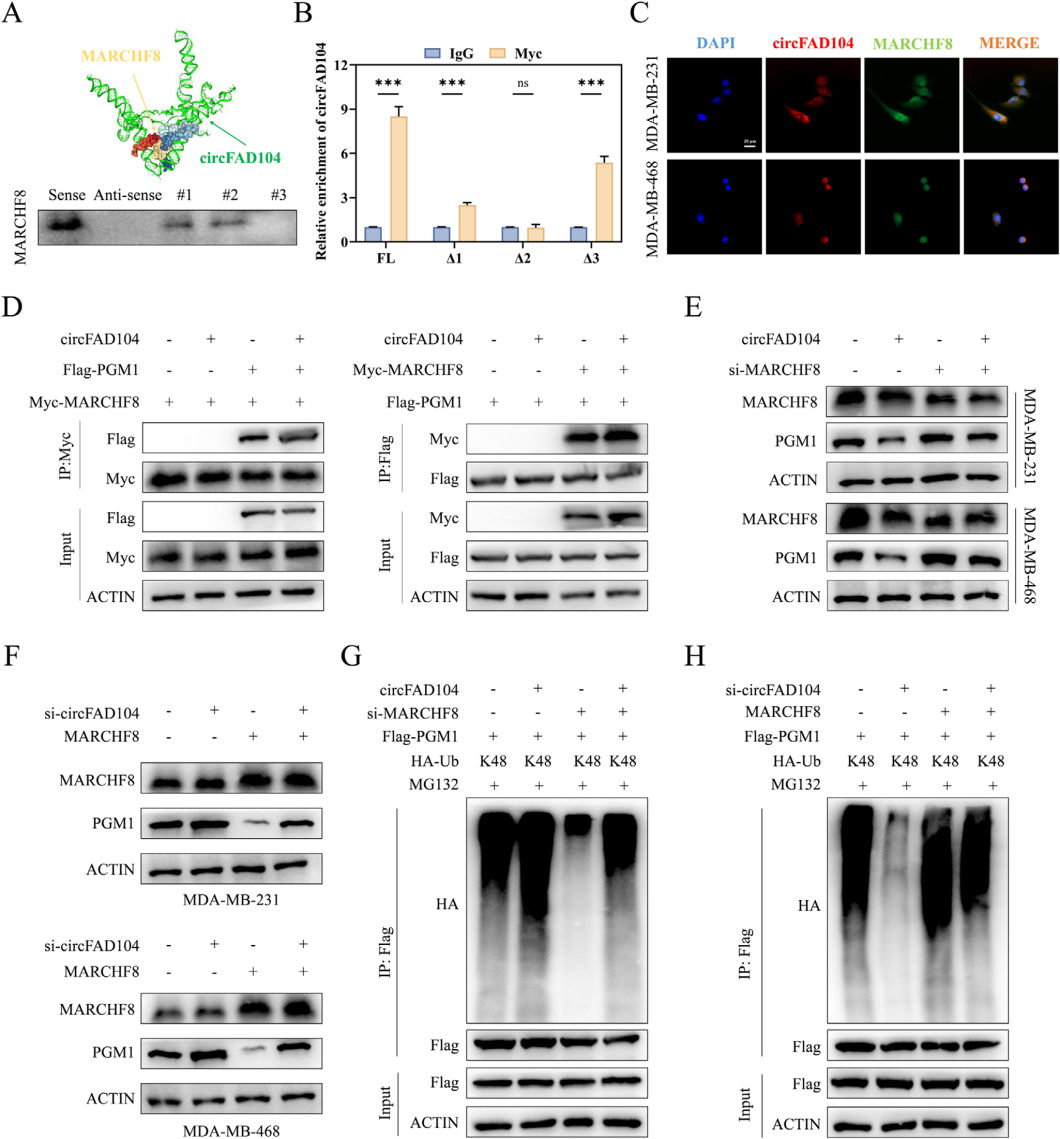
circFAD104 functions as a molecular scaffold to facilitate the MARCHF8‐PGM1 interaction. (A) RNA pull‐down assay using truncated circFAD104 probes to detect their binding to MARCHF8. (B) RIP assay using an anti‐Myc antibody to compare circFAD104 enrichment by full‐length MARCHF8 and MARCHF8 truncation mutants. (C) FISH‐IF colocalization assay of circFAD104 and MARCHF8 in TNBC cells. Scale bars, 20 µm. (D) Co‐IP assays showing the effect of circFAD104 overexpression on the MARCHF8‐PGM1 interaction. (E) Western blot analysis of PGM1 protein levels in TNBC cells with circFAD104 overexpression and MARCHF8 knockdown. (F) Western blot analysis of PGM1 protein levels in TNBC cells with circFAD104 knockdown and MARCHF8 overexpression. (G) Ubiquitination assay detecting PGM1 K48‐linked polyubiquitination after circFAD104 overexpression and MARCHF8 knockdown. (H) Ubiquitination assay detecting PGM1 K48‐linked polyubiquitination after circFAD104 knockdown and MARCHF8 overexpression. ns, not significant;***p < 0.001.

We then investigated how circFAD104 participates in the MARCHF8‐PGM1 interaction. CircFAD104 knockdown or overexpression did not alter MARCHF8 mRNA levels, protein abundance, or subcellular localization (Figure S28A–C), indicating that circFAD104 does not regulate MARCHF8 expression or localization. Molecular docking predicted a potential interaction between circFAD104 and MARCHF8. RNA pull‐down assay confirmed that MARCHF8 preferentially associated with the #1 and #2 fragments of circFAD104 (Figure 6A). RIP assays further demonstrated enrichment of circFAD104 with full‐length MARCHF8 and the RING‐TM1 region (Figure 6B). FISH‐IF analysis showed cytoplasmic colocalization of circFAD104 and MARCHF8 (Figure 6C, Figure S28D), supporting their spatial proximity. Exogenous and endogenous co‐IP experiments showed that circFAD104 overexpression enhanced the association between MARCHF8 and PGM1, whereas circFAD104 knockdown reduced this interaction (Figure 6D, Figure S28E–F). These findings indicate that circFAD104 facilitates the formation of a MARCHF8‐PGM1 E3 ligase‐substrate complex. To test whether MARCHF8 is required for circFAD104‐mediated PGM1 degradation, we performed rescue experiments. MARCHF8 knockdown reversed the decrease in PGM1 protein induced by circFAD104 overexpression (Figure 6E). Conversely, MARCHF8 overexpression abolished the PGM1 accumulation caused by circFAD104 knockdown (Figure 6F). Consistently, circFAD104‐induced K48‐linked ubiquitination of PGM1 was reduced by MARCHF8 knockdown (Figure 6G), whereas MARCHF8 overexpression restored PGM1 ubiquitination under circFAD104‐deficient conditions (Figure 6H). Collectively, these findings support a scaffold model in which circFAD104 bridges MARCHF8 and PGM1, thereby facilitating MARCHF8‐mediated K48‐linked ubiquitination and proteasomal degradation of PGM1.

### 3.7 CAF‐Derived Exosomal circFAD104 Promotes TNBC Progression in Vivo and Predicts Poor Clinical Outcomes

**FIGURE 7:**
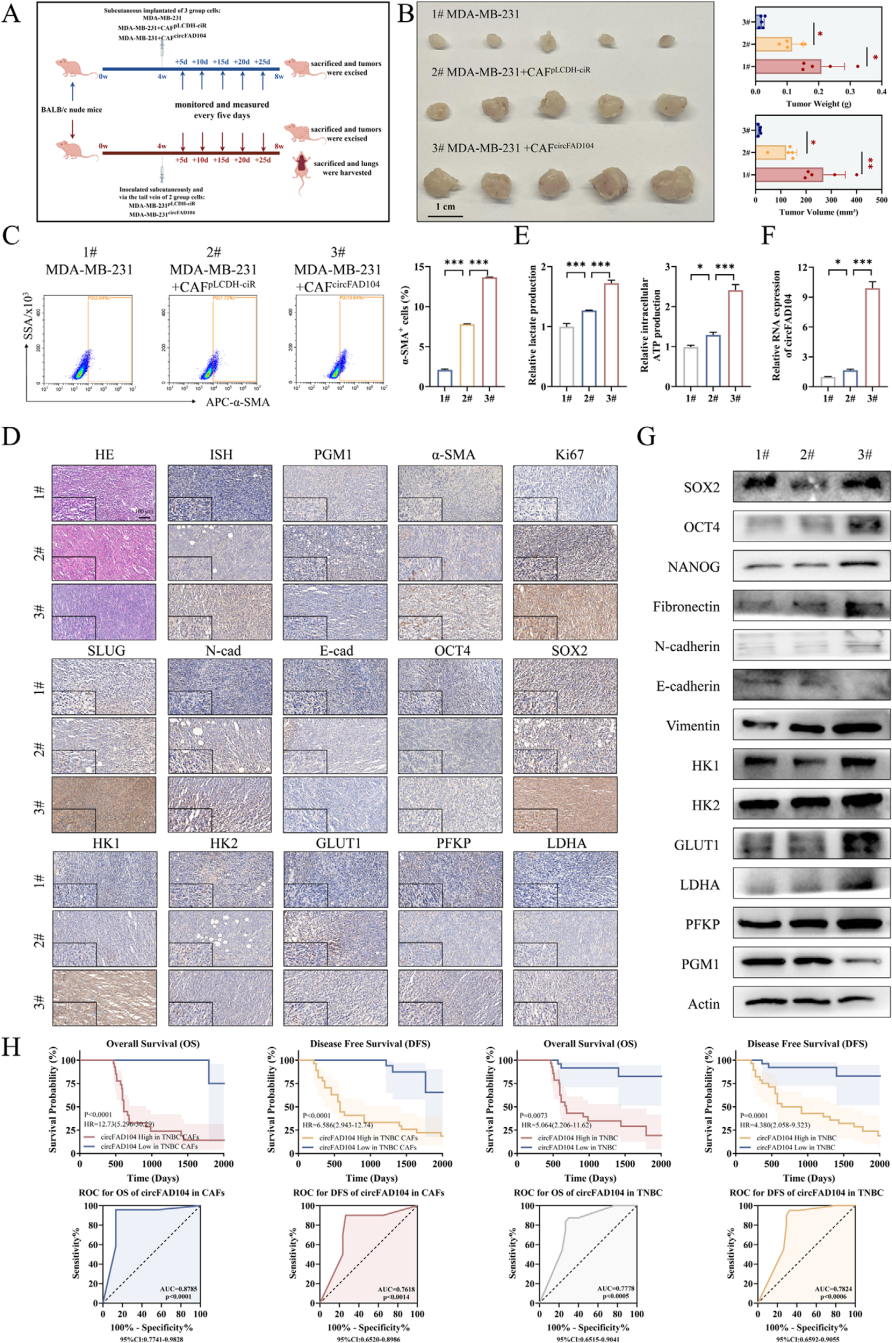
CAF‐derived exosomal circFAD104 promotes TNBC progression in vivo and predicts poor clinical outcomes. (A) Schematic of the subcutaneous xenograft model. (B) The tumor images, final tumor volume, and tumor weight in the indicated xenograft groups. Scale bars, 1 cm. (C) Flow cytometric analysis of α‐SMA^+^ CAF‐like cells in xenograft tissues. (D) Representative HE, ISH analysis of circFAD104, IHC staining of PGM1, Ki67, stemness‐related markers, EMT markers, and glycolysis‐related markers in xenograft tissues. Scale bars, 100 µm. (E) Lactate and ATP levels in xenograft tumor tissues. (F) qRT‐PCR analysis of circFAD104 expression in xenograft tissues. (G) Western blot analysis of PGM1, stemness‐related markers, EMT markers, and glycolysis‐related markers in xenograft tissues. (H) Kaplan‐Meier survival analysis and ROC curves evaluating the prognostic performance of circFAD104 expression in CAF‐enriched stromal and tumor‐cell compartments of TNBC tissues. *p < 0.05; **p < 0.01; ***p < 0.001.

We next assessed the in vivo impact of CAF‐derived circFAD104 on TNBC progression using a subcutaneous xenograft model in nude mice (Figure 7A). MDA‐MB‐231 cells were co‐injected with CAFs stably overexpressing circFAD104 (CAF^circFAD104^) or the control vector (CAF^pLCDH‐ciR^). A group receiving MDA‐MB‐231 cells alone served as an additional control. Co‐injection with CAF^circFAD104^ significantly accelerated tumor growth compared with the control groups, with increased tumor volume and tumor weight (Figure 7B). Flow cytometry of dissociated tumors showed increased α‐SMA^+^ CAF‐like populations in both CAF‐co‐injected groups (Figure 7C), a finding further supported by α‐SMA IHC staining (Figure 7D). Tumors from the CAF^circFAD104^ group exhibited higher lactate production and ATP levels, indicating enhanced glycolytic activity (Figure 7E). qRT‐PCR and ISH assays confirmed increased circFAD104 abundance in xenograft tissues from the CAF^circFAD104^ group, along with reduced PGM1 expression (Figure 7D,F,G). Consistently, tumors in the CAF^circFAD104^ group showed reduced E‐cadherin and increased mesenchymal, Ki67, glycolysis‐related markers, and stemness‐related markers (Figure 7D,G). These results indicate that CAF‐derived circFAD104 promotes TNBC tumor growth in vivo and recapitulates the metabolic and phenotypic reprogramming observed in vitro.

To further determine whether circFAD104 in tumor cells is sufficient to promote TNBC progression in vivo, we generated MDA‐MB‐231 cells stably overexpressing circFAD104 or the empty vector. Subcutaneous implantation showed that circFAD104 overexpression significantly promoted primary tumor growth (Figure S29A) and increased lactate production and ATP levels in xenograft tissues (Figure S29B). qRT‐PCR and ISH assays confirmed elevated circFAD104 expression in resected tumors (Figure S29C,D). IHC and Western blot analyses further showed that circFAD104 overexpression reduced PGM1 expression and increased EMT‐related, stemness‐related, and glycolysis‐related markers (Figure S29D,E). To assess metastatic potential, we established an experimental pulmonary metastasis model by intravenously injecting these cells into nude mice. Bioluminescent imaging and gross lung examination showed stronger lung metastatic signals and more visible metastatic nodules in mice injected with circFAD104‐overexpressing cells (Figure S29F,G). H&E staining confirmed more extensive pulmonary metastatic lesions in this group (Figure S29H). Together, these findings show that tumor‐cell circFAD104 overexpression promotes tumor growth and pulmonary metastasis in vivo.

Clinically, circFAD104 expression was evaluated in tumor tissues from 54 TNBC patients in the discovery cohort. The optimal cutoff value for circFAD104 expression in tumor‐cell and CAF‐enriched stromal compartments was determined by ROC curve analysis based on survival status. Using this cutoff, patients were stratified into circFAD104‐high and circFAD104‐low groups. Elevated circFAD104 expression in either compartment was associated with distant metastasis (Figure S30A, Table S1). Patients with high circFAD104 expression exhibited significantly shorter overall survival and disease‐free survival than those with low circFAD104 expression (Figure 7H). To validate these clinical associations, we analyzed an independent cohort of 95 TNBC patients, in which circFAD104 was scored separately in tumor‐cell and CAF‐enriched compartments using the same ISH strategy. In this validation cohort, high circFAD104 expression in either compartment was significantly associated with lymph‐node metastasis and distant metastasis (Figure S30B, Table S2). Kaplan‐Meier and ROC analyses further confirmed the prognostic value of circFAD104 expression in both compartments (Figure S30C). Collectively, these data demonstrate that circFAD104 promotes TNBC progression and predicts poor clinical outcomes, supporting the clinical relevance of the CAF‐derived exosomal circFAD104‐PGM1 metabolic axis.

## 4 Discussion

Breast cancer is the most prevalent malignant tumor among women worldwide, with metastasis accounting for the majority of cancer‐related deaths [35]. Among its molecular subtypes, TNBC exhibits a high recurrence rate, aggressive metastatic potential, and poor clinical outcomes, presenting a critical clinical challenge [2, 36]. Mounting evidence indicates that tumor progression is not solely dictated by cancer cells but is profoundly shaped by dynamic crosstalk within the TME. For instance, extracellular matrix (ECM) remodeling enhances cancer stemness and EMT [37], while metabolic interactions, such as lactate‐driven acidification, suppress anti‐tumor immunity [38]. Moreover, stromal‐derived signaling molecules, including exosome‐carried non‐coding RNAs and cytokines, can directly promote tumor metastasis [39, 40]. As the predominant stromal cell population in the TME [41], CAFs exert multifaceted influences on cancer biology. While prior studies have established that CAFs modulate tumor behavior via exosomal non‐coding RNAs, the role of CAFs in regulating metabolic reprogramming of TNBC cells through exosomal circRNAs remains poorly understood.

Aerobic glycolysis, a hallmark of metabolic reprogramming, underpins the aggressive proliferation, invasiveness, and metastatic potential of tumors [42]. Among breast cancer subtypes, TNBC exhibits heightened dependence on ATP and metabolic intermediates generated by glycolysis to sustain its malignant phenotype [43]. While dysregulation of metabolic enzymes within tumor cells has been extensively studied, the role of CAFs, particularly their capacity to reshape glucose flux via exosome‐mediated transfer of non‐coding RNAs, remains poorly understood. This represents a critical gap in deciphering TNBC's metabolic vulnerabilities. CAFs are known to promote malignancy through diverse secreted factors [44]. For instance, CAF‐derived SPP1 enhances resistance to tyrosine kinase inhibitors and promotes EMT in hepatocellular carcinoma [45], while CAF‐secreted POSTN promotes papillary thyroid tumor growth through activation of integrin‐FAK‐STAT3 signaling [46]. Consistent with these findings, our data show that CAF‐conditioned medium (CM) significantly enhances TNBC cell proliferation and motility. Beyond soluble factors, exosomes serve as important vehicles for intercellular communication by delivering bioactive molecules, including circRNAs, which are increasingly recognized as key regulators of tumor biology. Although several circRNAs have been implicated in breast cancer, such as circRPPH1, which promotes progression through FAK/PI3K/AKT activation [47], and circSKA3, which induces invadopodia formation by enhancing Tks5/integrin β1 co‐localization [48], whether CAF‐derived exosomal circRNAs directly regulate TNBC metabolic reprogramming remains largely unknown. To address this, we performed RNA sequencing of exosomes isolated from patient‐derived CAFs and matched NFs. Among the differentially expressed circRNAs, circFAD104 was markedly upregulated in both CAFs and CAF‐derived exosomes, and PKH26‐labeled uptake assays confirmed that TNBC cells efficiently internalize these exosomes. Functionally, circFAD104 enhanced TNBC stemness, metastatic traits, and glycolytic activity, whereas glycolytic inhibition by 2‐DG attenuated these phenotypes. These findings position circFAD104 as a CAF‐derived exosomal effector that links stromal communication to metabolic and phenotypic reprogramming in TNBC.

To delineate the molecular mechanisms underlying circFAD104‐driven metabolic reprogramming, we performed RNA pull‐down followed by mass spectrometry. Among the identified interactors, PGM1 emerged as a key candidate because of its strong circFAD104‐binding signal and its central role in carbon metabolism. PGM1 catalyzes the reversible interconversion of G1P and G6P, serving as a critical node linking glycogen synthesis, glycolysis, and galactose metabolism. Growing evidence highlights the context‐dependent roles of PGM1 in cancer. For example, PGM1 acts as a tumor suppressor in hepatocellular carcinoma via FOXJ2‐mediated glucose transport regulation and in colorectal cancer by suppressing EMT [31, 49], yet paradoxically supports cell survival in lung cancer through the AMPK‐HDAC8 axis and in glioma via Myc‐driven bioenergetics [32, 50]. Its role in TNBC, however, has remained poorly defined. Here, bioinformatic analyses showed that PGM1 is downregulated in breast cancer at the bulk level while exhibiting cell‐type‐ and spatial‐context‐dependent distribution within TNBC tissues. Functionally, PGM1 knockdown enhanced TNBC cell proliferation, stemness, migration, invasion, and glycolytic activity, whereas PGM1 overexpression suppressed these phenotypes. Importantly, PGM1 overexpression also reduced subcutaneous tumor growth and pulmonary metastatic burden in vivo, supporting its tumor‐suppressive function in TNBC.

Because the RNA pull‐down/MS analysis also identified other glycolysis/gluconeogenesis‐related candidates, including PDHB and GALM, we evaluated whether these proteins contribute to the circFAD104‐mediated phenotype. PDHB showed some effects on migration and lactate production, consistent with its known role in pyruvate oxidation, whereas GALM manipulation produced no consistent effects on migration, ATP production, or lactate production. However, RIP assays showed that circFAD104 was not enriched by either PDHB or GALM, suggesting that their presence in the pull‐down might be due to indirect interactions within large protein complexes rather than direct scaffolding. These results do not exclude circFAD104‐independent roles of PDHB or GALM in TNBC metabolism, but they support prioritization of PGM1 as the direct downstream effector in the mechanism described here. Mechanistically, circFAD104 overexpression markedly reduced PGM1 protein abundance without altering PGM1 mRNA levels, resulting in increased ECAR, glucose consumption, lactate production, ATP, and G6P, along with decreased G1P. Conversely, PGM1 restoration attenuated circFAD104‐induced malignant and metabolic phenotypes. Collectively, these data establish PGM1 as a tumor‐suppressive metabolic regulator in TNBC and demonstrate that circFAD104 exerts its oncogenic function, at least in part, through post‐translational suppression of PGM1, specifically by promoting its degradation rather than altering its transcription.

Given that circFAD104 reduced PGM1 protein levels without affecting its mRNA, we hypothesized that PGM1 is regulated post‐translationally. Treatment with the proteasome inhibitor MG132 rescued PGM1 abundance in circFAD104‐overexpressing cells, implicating the ubiquitin‐proteasome system. To identify the responsible E3 ligase, we integrated E3 ligase prediction, molecular docking, and molecular dynamics simulation, which prioritized MARCHF8, a RING‐domain‐containing E3 ligase, as a PGM1‐interacting protein. MARCHF8 overexpression reduced PGM1 protein levels, whereas MARCHF8 knockdown increased PGM1 abundance and prolonged its half‐life. Ubiquitination assays further showed that MARCHF8 promotes K48‐linked, but not K63‐linked, polyubiquitination of PGM1. RNA pull‐down and RIP assays demonstrated that circFAD104 binds both MARCHF8 and PGM1, supporting a scaffold model in which circFAD104 nucleates a MARCHF8‐PGM1 E3 ligase‐substrate complex. Mapping experiments localized the interaction to the 1–190 and 191–354 nt regions of circFAD104, the D3/D4 domains of PGM1, and the RING‐TM1 region of MARCHF8. Co‐IP and rescue assays further confirmed that circFAD104 enhances the MARCHF8‐PGM1 association and promotes MARCHF8‐dependent K48‐linked ubiquitination and proteasomal degradation of PGM1. Together, these findings establish circFAD104 as a molecular scaffold that drives MARCHF8‐dependent degradation of PGM1, thereby disrupting the G1P/G6P equilibrium and promoting a glycolytic shift in TNBC cells.

MARCHF8 has been primarily studied in viral immunity and inflammation [51, 52], but emerging evidence reveals its context‐dependent roles in cancer. It can inhibit fatty acid synthesis and lipid accumulation by degrading SREBP1 to suppress hepatocellular carcinoma progression [53], and it suppresses glycolysis in colorectal cancer through ubiquitination of HK2 [54]. Conversely, MARCHF8 can act oncogenically, promoting hepatocellular carcinoma progression by degrading PTEN [55], or facilitating pancreatic cancer metastasis via PTPN4 degradation [56]. Here, we identify PGM1 as a previously unrecognized substrate of MARCHF8 in TNBC. This pro‐tumorigenic function contrasts with the glycolysis‐suppressive role of MARCHF8 in colorectal cancer, underscoring how cancer‐type‐specific contexts may determine substrate selection and functional outcome. Thus, an E3 ligase can exert divergent effects on tumor metabolism depending on tissue‐specific metabolic dependencies and substrate availability. Further work is warranted to delineate how MARCHF8 selects and regulates metabolic enzymes across cancer types.

It should be noted that several concepts addressed here have been previously reported, such as CAF‐derived exosomal circRNAs, circRNA scaffold functions, tumor‐suppressive roles of PGM1 in certain contexts, and the pleiotropic functions of MARCHF8. Several CAF‐derived exosomal circRNAs, such as circEIF3K [19], circIFNGR2 [20], and circTBPL1 [21], promote tumor progression through miRNA sponge mechanisms. Other scaffold circRNAs such as circSKA3, which bridges integrin β1 and TKS5 to drive invadopodia formation [57], and circIPO11, which engages STK3 to regulate Hippo signaling [58], act on structural or kinase partners. CircFAD104 differs from both groups in three respects. First, although delivered by CAF‐derived exosomes, it functions as a protein scaffold rather than primarily as a miRNA sponge. Second, it physically bridges an E3 ubiquitin ligase (MARCHF8) and a metabolic tumor suppressor (PGM1) to promote a specific K48‐linked ubiquitination event, an enzyme‐substrate pairing not previously reported. Third, the downstream consequence is metabolic, with this scaffold‐mediated ubiquitination redirecting glucose‐phosphate metabolism toward glycolysis in TNBC. Even relative to known scaffold circRNAs, therefore, circFAD104 engages an entirely distinct partner set and operates in a different pathway. In this study, we characterized the tumor‐suppressive role of PGM1, identified it as a MARCHF8 substrate, and established CAF‐derived exosomal circFAD104 as an upstream regulator in TNBC. This represents a context‑specific mechanistic integration that mediates the communication between CAFs and TNBC cells.

The clinical relevance of circFAD104 in TNBC is supported by analyses of both a discovery cohort and an independent validation cohort. In the discovery cohort of 54 TNBC patients, elevated circFAD104 expression in tumor‐cell or CAF‐enriched stromal compartments showed a nominal association with distant metastasis and was associated with markedly shorter overall and disease‐free survival. To strengthen this evidence, we analyzed an independent validation cohort of 95 TNBC patients using the same compartment‐specific ISH scoring strategy. High circFAD104 expression in either compartment was significantly associated with poor clinical outcome. These findings support the prognostic significance of circFAD104 while highlighting the need for larger, multi‐center cohorts with longer follow‐up. The dual‐compartment distribution of circFAD104 further suggests that the tumor microenvironment harbors prognostic information beyond malignant cells alone. Consistent with this, infiltration by SLC14A1^+^ CAFs correlates with cancer stemness, poor therapeutic response, and worse outcomes in bladder cancer [59]. Moreover, CAF‐derived exosomal non‐coding RNAs are emerging as liquid and tissue‐based biomarkers. Plasma exosomes enriched in CAF‐secreted circSTAT3 were associated with lymph node metastasis in TNBC [23], while CAF‐released exosomal miR‐196a promotes cisplatin resistance and correlates with overall survival in head and neck cancer [60]. Our findings align with this evolving paradigm, positioning CAF‐enriched circFAD104 as a stroma‐informed prognostic indicator that reflects active metabolic reprogramming within the TME.

Therapeutically, the targeting of oncogenic circRNAs is advancing rapidly. Antisense oligonucleotides (ASOs) have silenced circSKA3 to restore chemosensitivity in colorectal cancer [57], and CRISPR/Cas9‐mediated knockout of circIPO11 impaired liver cancer stem cell self‐renewal and suppressed hepatocellular carcinoma progression [58]. These studies provide a strong rationale for developing circFAD104‐specific ASOs or CRISPR‐based tools to disrupt its tumor‐promoting functions in TNBC. Beyond direct circRNA inhibition, our identification of the MARCHF8‐PGM1 ubiquitination‐glycolysis axis opens additional avenues for metabolic intervention. Recent work in bladder cancer demonstrated that combined targeting of the ubiquitin‐conjugating enzyme UBE2C and glutamine metabolism (via CB‐839) effectively suppresses lymphangiogenesis and lymph node metastasis by disrupting the UBE2C/SNAT2/glutamine/VEGFC axis [61]. By analogy, small molecules or peptide‐based strategies that disrupt the MARCHF8‐PGM1 interaction, or agents targeting downstream glycolytic dependence, may reverse circFAD104‐driven glycolytic reprogramming and impair TNBC stemness and metastasis. These therapeutic implications nonetheless remain preliminary and require extensive validation in clinically relevant models.

## 5 Conclusions

**FIGURE 8:**
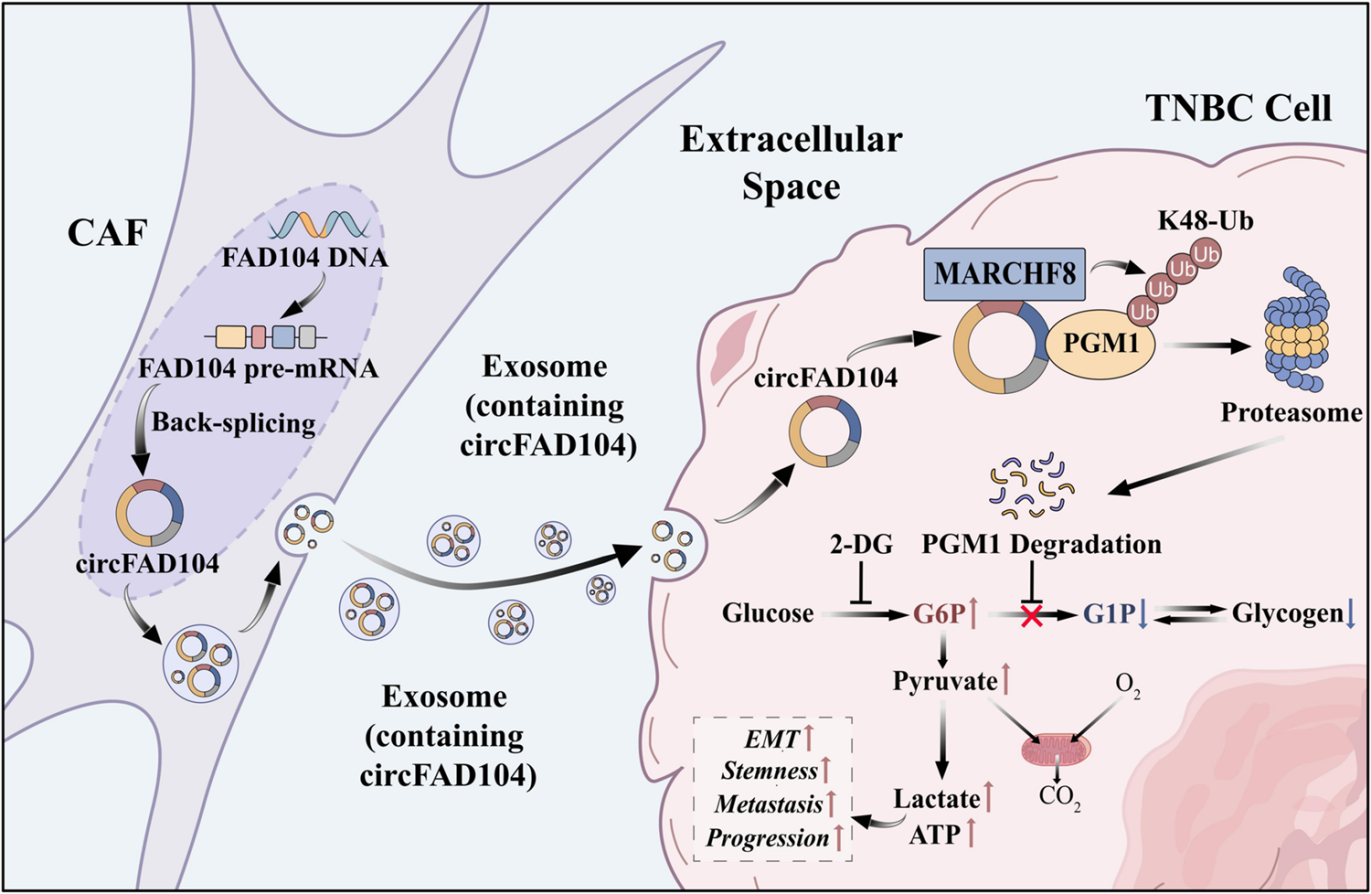
Schematic illustration of the proposed mechanism by which CAF‐derived exosomal circFAD104 promotes TNBC progression through the MARCHF8/PGM1 axis and glycolytic reprogramming. CircFAD104 facilitates the interaction between MARCHF8 and PGM1, enhancing MARCHF8‐dependent K48‐linked ubiquitination and proteasomal degradation of PGM1 and thereby shifting glucose‐phosphate metabolism toward glycolysis.

In summary, this study identifies CAF‐derived exosomal circFAD104 as a stromal circRNA that promotes TNBC metabolic reprogramming and progression (Figure 8). Mechanistically, circFAD104 functions as a molecular scaffold that facilitates the MARCHF8‐PGM1 interaction, leading to K48‐linked ubiquitination and proteasomal degradation of PGM1. Loss of PGM1 shifts glucose‐phosphate metabolism toward glycolysis, thereby enhancing TNBC stemness, EMT, migration, invasion, tumor growth, and pulmonary metastasis; these phenotypes are reversible by glycolytic inhibition or PGM1 restoration. Clinically, circFAD104 expression in tumor‐cell and CAF‐enriched compartments predicts poor outcomes in TNBC. Several limitations remain. First, more specific surface or functional markers for CAF subtypes will be needed to precisely isolate and spatially map the CAF populations responsible for circFAD104 delivery. Second, although MARCHF8‐PGM1 is a key downstream axis, circRNAs can modulate multiple pathways, and additional circFAD104 targets may exist. Third, while the validation cohort strengthens our clinical conclusions, larger multi‐center cohorts with more distant‐metastasis events and longer follow‐up are required to fully establish its prognostic and predictive value. Addressing these questions may facilitate the development of circFAD104‐based biomarkers and therapeutic strategies for TNBC.

## Author Contributions

L.W., X.L., and Q.Y. were responsible for the conception and design of the study. L.W., X.L., Y.L., Y.W., Y.J., J.W., D.L., Y.Y., and J.Y. carried out the data acquisition. L.W., X.L., Y.L., Y.W., Y.J., J.W., D.L., Y.Y., J.Y., Y.L., T.C., D.H., and Z.W. performed the analysis and interpretation of the data. W.Z., B.C., L.W., and Q.Y. oversaw the study. L.W., X.L., and Q.Y. wrote, reviewed, and revised the manuscript. All authors read and approved the final manuscript.

## Funding

This work was supported by Special Foundation for Taishan Scholars (No. tstp20250509, tsqn202507348), National Natural Science Foundation of China (No. 82573211, 82373005, 82573252), Postdoctoral Innovation Program of Shandong Province (No. SDCX‐ZG‐202501049).

## Conflicts of Interest

The authors declare no conflicts of interest.

## Supporting information




**Supporting File**: advs77504‐sup‐0001‐SuppMat.docx.

## Data Availability

The data that support the findings of this study are available from the corresponding author upon reasonable request.
