# Supplementary material for: Cancer‐Associated Fibroblasts Promote Glucose Metabolic Reprogramming and Progression of Triple‐Negative Breast Cancer via Exosomal circFAD104‐Mediated Intercellular Communication
Source: Adv Sci (Weinh). 2026 Aug 29:e77504. Online ahead of print. doi: 10.1002/advs.77504 (PMC13525701; doi:10.1002/advs.77504)
Supplement: Supplementary file 1 [file ADVS-9999-e77504-s001.docx]

**Supplementary Materials**

**Title:** Cancer-associated fibroblasts promote glucose metabolic reprogramming and progression of triple-negative breast cancer via exosomal circFAD104-mediated intercellular communication

**Running Title:** CAF-derived circFAD104 promotes TNBC progression

Lei Wang^1, #^, Xiaoyan Li^1, #^, Yiran Liang^1^, Yifei Wang^1^, Yuhan Jin^1^, Jianing Wang^1^, Dan Luo^1^, Yuxia Yang^1^, Jingze Yang^1^, Yaming Li^1^, Tong Chen^1^, Dianwen Han^1^, Zekun Wang^1^, Wenjing Zhao^2^, Bing Chen^2^, Lijuan Wang^2^, and Qifeng Yang^1, 2, 3, *^

**Authors’ affiliations:**

^1^Department of Breast Surgery, General Surgery, Qilu Hospital of Shandong University, Jinan, Shandong, 250012, P.R. China.

^2^Biological Resource Center, Qilu Hospital of Shandong University, Jinan, Shandong, 250012, P.R. China.

^3^Research Institute of Breast Cancer, Shandong University, Jinan, Shandong, 250012, P.R. China.

#These authors contributed equally to this work.

***Correspondence to:**

Qifeng Yang, Qilu Hospital of Shandong University, Wenhua Xi Road No. 107, Jinan, Shandong, 250012, China; Tel: 86-18560085168; E-mail: qifengy_sdu@163.com

**Supplementary Materials and Methods**

**Cell Culture**

TNBC cell lines MDA-MB-231 (RRID: CVCL_0062), MDA-MB-468 (RRID: CVCL_0419), and HEK293T cells were obtained from the American Type Culture Collection (ATCC, Manassas, VA, USA) and cultured in DMEM (Macgene Biotech, Beijing, China) supplemented with 10% FBS at 37°C in a humidified incubator with 5% CO₂.

**Plasmid Construction, Transfection and Lentiviral Transduction**

Full-length PGM1 and MARCHF8 coding sequences as well as the indicated truncated mutants were cloned into mammalian expression vectors with N-terminal FLAG or Myc tags. PGM1 truncation mutants included Δ1-Δ4 (Δ1, D1; Δ2, D2; Δ3, D3; Δ4, D4) and the D3-D4 fragment, and MARCHF8 truncation mutants included Δ1-Δ3 (Δ1, RING; Δ2, TM1+TM2; Δ3, RING+TM1). Plasmids and small interfering RNAs (siRNAs; GenePharma, Shanghai, China, sequences provided in Table S4) were transiently transfected using jetPRIME reagent (Polyplus-transfection, Illkirch, France) according to the manufacturer’s instructions. Stable cell lines were generated by lentiviral transduction followed by puromycin selection for 3-4 weeks.

**RNA Extraction and Quantitative Real-Time PCR (qRT-PCR)**

Total RNA was extracted from cells, exosomes, or transfected/exosome-treated cells using TRIzol reagent (Invitrogen, Carlsbad, CA, USA). Briefly, 200 μL of chloroform was added to 1 mL of TRIzol, mixed by shaking, and incubated for 10 minutes at room temperature. Subsequently, the upper aqueous phase was collected following centrifugation at 12,000×g for 15 minutes at 4°C. RNA was precipitated with an equal volume of isopropanol, washed with 75% ethanol prepared in DEPC water, and finally dissolved in 20 μL of DEPC water. The concentration of RNA was measured using a Nanodrop spectrophotometer (Thermo Fisher Scientific, Waltham, MA, USA) to assess its quality. For qRT-PCR, complementary DNA (cDNA) was synthesized using the PrimeScript Reverse Transcriptase (RT) Reagent Kit (TaKaRa) specifically for mRNA and circRNA reverse transcription. The qRT-PCR was conducted using SYBR Premix Ex Taq I (TaKaRa) along with gene-specific primers (sequences provided in Table S5). β-actin served as the internal control for normalizing mRNA and circRNA expression levels. The relative expression levels were determined using the 2⁻^ΔΔCT^ method.

**RNase R Treatment and Actinomycin D (ActD) Assay**

To verify the circular nature and stability of circFAD104, 2 μg of total RNA was incubated with 3 U of RNase R (Epicentre, Madison, WI, USA) at 37°C for 20 minutes, followed by inactivation at 70°C for 10 minutes. A parallel group without RNase R treatment served as the control. For RNA half-life analysis, cells were treated with 5 μg/mL of ActD (Sigma-Aldrich, St. Louis, MO, USA) for 0, 4, 8, 12, or 24 hours to inhibit de novo RNA synthesis. Subsequently, total RNA was extracted at each time point to evaluate the expression levels of circFAD104 and its linear host gene, FAD104 mRNA, via qRT-PCR.

**Western Blot**

Proteins were extracted from transfected cells, exosome-treated cells, or exosomes using Cell Lysis Buffer for Western blotting and immunoprecipitation (Beyotime, Shanghai, China), supplemented with phenylmethylsulfonyl fluoride (PMSF) and sodium fluoride (NaF) to inhibit protease and phosphatase activity. The protein concentration was quantified using the BCA Protein Assay Kit (Millipore, Burlington, MA, USA) to ensure equal loading. Proteins were separated by 10% SDS-PAGE and transferred onto 0.22 μm PVDF membranes (Millipore, Burlington, MA, USA). The membranes were blocked with 5% non-fat milk in 1×TBST for 1 hour at room temperature, followed by incubation with specific primary antibodies (details listed in Table S3) overnight at 4°C. After three washes with 1×TBST (5 minutes each), the membranes were incubated with HRP-conjugated secondary antibodies for 1 hour at room temperature, followed by three additional washes with 1×TBST to remove non-specific binding. Target protein signals were visualized using the ECL chemiluminescence detection system (Bio-Rad, Hercules, CA, USA), with β-actin employed as the internal loading control for normalization.

**Glucose Consumption, Glucose-6-Phosphate (****G6P) and Glucose-1-Phosphate (G1P) Assays**

Glucose consumption, intracellular G6P and G1P levels were measured using commercial assay kits according to the manufacturers’ instructions. Glucose levels in culture supernatants were measured using the Glucose Assay Kit with O-toluidine (Beyotime, Shanghai, China). Intracellular G6P levels were determined from cell lysates using the G6P Assay Kit with WST-8 (Beyotime, Shanghai, China). Intracellular G1P levels were measured from cell lysates using a G1P assay kit from Jianglai Biotechnology (Shanghai, China). Values were normalized to total protein concentration and are presented as relative levels.

**MTT Assay**

Transfected or exosome-treated TNBC cells were seeded into 96-well plates at a density of 1.5 × 10³ cells per well and cultured under standard conditions. After 24 h of attachment, cells were treated with vehicle control or 10 mM 2-deoxy-D-glucose (2-DG; Sigma-Aldrich, USA; Cat. No. D6134) and maintained for the entire duration of the assay. At specified time points, 20 μL of MTT solution (5 mg/mL) was added to each well, followed by incubation for an additional 4 to 6 hours at 37°C. The supernatant was then carefully aspirated, and 100 μL of dimethyl sulfoxide (DMSO) was added to dissolve the formazan crystals. The optical density (OD) at 490 nm was measured using a microplate reader (Bio-Rad, Hercules, CA, USA), and a proliferation curve was generated based on the OD values.

**EdU Incorporation Assay**

Cell proliferation was further evaluated using the EdU Incorporation Assay Kit (RiboBio, Guangzhou, China). TNBC cells (1 × 10⁴ cells per well) were seeded into 96-well plates one day before to ensure adherence. Then the culture medium was replaced with vehicle control or 10 mM 2-DG, and cells were cultured for another 24 h. The cells were incubated with the EdU working solution (prepared according to the kit instructions) for 2 hours, subsequently fixed with 4% paraformaldehyde for 20 minutes, and permeabilized with 0.5% Triton X-100 for 15 minutes at room temperature. Following blocking, the cells were stained with Apollo dye for 1 hour, and nuclei were counterstained with Hoechst for 30 minutes in the dark. EdU-positive cells were visualized and enumerated using a fluorescence microscope (Olympus, Japan).

**Colony Formation Assay**

Transfected TNBC cells (1 × 10³ cells per dish) were seeded into 60-mm cell culture dishes and cultured for 2-3 weeks under standard conditions, with the medium changed every 3-4 days. For 2-DG treatment, vehicle control or 10 mM 2-DG was added after cell attachment and refreshed with each medium change. Colonies were washed three times with 1 × PBS, fixed with methanol for 15 minutes at room temperature, and stained with a 0.5% crystal violet solution for an additional 15 minutes. The colonies were imaged and manually counted for statistical analysis.

**Transwell Assay**

Transwell chambers (8 μm pore size; Corning Costar, Corning, NY, USA) were placed in 24-well plates, and 700 μL of DMEM supplemented with 20% FBS was added to the lower compartment. For migration assays, 6-8 × 10⁴ TNBC cells (either transfected or treated with exosomes) were resuspended in 200 μL of serum-free DMEM and added into the upper compartment. For invasion assays, the upper compartment was pre-coated with 60 μL of Matrigel (BD Biosciences, San Jose, CA, USA) and incubated at 37°C for 30 minutes to establish a basement membrane-like layer before cell seeding. For 2-DG treatment, vehicle control or 10 mM 2-DG was added to the upper chambers at the time of cell seeding and maintained for 24-48h. After incubation for 24-48 hours, cells on the upper surface of the membrane were gently removed with a cotton swab. Cells adhered to the lower surface were washed with 1× PBS, fixed with methanol for 15 minutes, and stained with 0.5% crystal violet for 15 to 20 minutes. The stained cells were imaged using a light microscope.

**Wound Healing Assay**

Transfected or exosome-treated TNBC cells were seeded into 24-well plates and cultured until reaching 90% confluence. A sterile 10 μL pipette tip was used to create a straight wound across the cell monolayer, after which the remaining cells were washed three times with 1× PBS to remove debris. The cells were then cultured in serum-free DMEM to eliminate the influence of serum on proliferation. For 2-DG treatment, vehicle control or 10 mM 2-DG was added immediately after scratching and maintained for the duration of the wound closure assay. Wound closure was documented by capturing images at 0 h and 48 h using a light microscope (Olympus, Japan).

**Tube Formation Assay**

HUVECs were used for tube formation assays. Transfected cells were cultured in serum-free DMEM, and the medium was collected as conditioned medium 24 hours post-transfection. The collected medium was centrifuged at 3000×g for 15 minutes at 4°C to eliminate cell debris. Subsequently, 5×10⁴ HUVECs were resuspended in the processed conditioned medium and plated onto 48-well plates pre-coated with 150 μL of Matrigel (BD Biosciences, San Jose, CA, USA). After 4 to 6 hours of incubation, the formed capillary tube structures were imaged under a light microscope, and the tube length was measured for quantification.

**Sphere Formation Assay**

Transfected or exosome-treated TNBC cells were seeded into Ultra-Low Attachment 96-well plates at a density of 500 cells per well and cultured in DMEM/F12 medium supplemented with 20 ng/mL human EGF, 20 ng/mL fibroblast growth factor, B27 (1:50), 5 μg/mL insulin, 100 U/mL penicillin, and 100 μg/mL streptomycin. For 2-DG treatment, vehicle control or 10 mM 2-DG was added after seeding and refreshed with each medium change. After 5-7 days of cultivation, formed spheres were imaged using a light microscope, and their diameters were measured to assess the sphere-forming capacity.

**Extreme Limiting Dilution Analysis (ELDA)**

TNBC cells were seeded into Ultra-Low Attachment 96-well plates at gradient densities (1000, 100, 50, or 10 cells/well) using the same specific medium as the sphere formation assay. After culturing to allow sphere formation, the number of wells with spheres was recorded, and tumor-initiating cell frequency was calculated using ELDA software (wehi.edu.au), a tool for limiting dilution analysis in stem cell research.

**Phalloidin Staining**

TNBC cells were seeded on cell slides in advance to allow adherence. After treatment, cells were washed with 1×PBS, fixed with 4% paraformaldehyde, and permeabilized with 0.2% Triton X-100. The cytoskeleton was stained with Phalloidin dye (Thermo Fisher Scientific, Waltham, MA, USA) diluted at 1:60, and cell nuclei were counterstained with DAPI. Stained cells were observed and imaged using a fluorescence microscope to assess cytoskeletal changes.

**Collection of Conditioned Medium (CM)**

To collect secreted substances from transfected CAFs, the culture medium of transfected CAFs was replaced with serum-free DMEM at 48 h post-transfection. After continued culture for 24 h, the medium was harvested and centrifuged at 3000×g for 15 min at 4°C to remove cell debris; the supernatant was collected as CAF-derived CM for subsequent breast cancer cell treatment.

**Hematoxylin-Eosin (HE) Staining**

HE staining was conducted to examine the morphological characteristics of tumor tissues, utilizing the HE Stain Kit (Solarbio, Beijing, China). In brief, paraffin-embedded sections were deparaffinized, then stained with hematoxylin to stain nuclei and eosin to stain the cytoplasm. Following staining, the sections were dehydrated and sealed. The stained sections were subsequently imaged using a light microscope.

**Immunohistochemistry (IHC)**

Paraffin-embedded tumor tissues were sectioned into 4 μm thick slices. The sections were deparaffinized, rehydrated, and subjected to antigen retrieval using EDTA antigen retrieval solution. Endogenous peroxidase activity was blocked with 3% hydrogen peroxide, and sections were blocked with 5% bovine serum albumin (BSA). The sections were then incubated with the indicated primary antibodies overnight at 4°C, followed by horseradish peroxidase (HRP)-conjugated secondary antibodies. Signals were visualized using 3,3'-diaminobenzidine (DAB) solution (ZSGB-BIO, Beijing, China), and nuclei were counterstained with hematoxylin. Images were acquired using a light microscope (Olympus, Japan). The primary antibodies used for IHC are listed in Table S3.

**Supplementary Figures**

**Fig. S1 Phenotypic validation of CAFs and NFs.** (A) Immunofluorescence staining of Vimentin, fibroblast activation protein (FAP), α-smooth muscle actin (α-SMA), and fibroblast-specific protein 1 (FSP-1) in NFs and CAFs; nuclei stained with DAPI. Scale bars, 10 μm. (B) Western blot analysis of Vimentin, α-SMA, FAP, and FSP-1 protein levels in NFs and CAFs; β-actin as loading control. Quantification of IF fluorescence intensity and Western blot band intensity is shown adjacent to the representative images.

**Fig. S2 Characterization of exosomes and circFAD104 expression.** (A) Western blot analysis of exosomal markers (CD9, CD63) and non-exosomal markers (Calnexin, GM130) in NF/CAF-derived exosomes and parental cells. (B) Transmission electron microscopy (TEM) images of NF-EXO and CAF-EXO. (C) Nanoparticle tracking analysis (NTA) of NF-EXO and CAF-EXO size distribution. (D) Fluorescence microscopy images of TNBC cells incubated with PKH26-labeled exosomes (red); nuclei stained with DAPI. Scale bars, 50 μm. (E) qRT-PCR analysis of circFAD104 expression in CAFs, TNBC cells, and other breast cancer cells.

**Fig. S3 circFAD104 knockdown in CAFs abrogates pro-tumorigenic effects.** (A) Schematic of siRNA targeting the back-splicing junction of circFAD104. (B) qRT-PCR validation of circFAD104 and linear FAD104 mRNA levels in CAFs transfected with si-NC or si-circFAD104. (C) EdU assay of TNBC cell proliferation treated with conditioned medium (CM) from NFs, si-NC-transfected CAFs, or si-circFAD104-transfected CAFs. Scale bars, 200 μm. (D) Transwell assay of TNBC cell migration treated with CM from different groups. Scale bars, 200 μm. ns, not significant; *P < 0.05; **P < 0.01; ***P < 0.001.

**Fig. S4 CAF-2-derived exosomal circFAD104 promotes TNBC cell EMT and metastasis.** (A) Table for group serial number labeling. (B) qRT-PCR analysis of circFAD104 levels in TNBC cells post-exosome treatment. (C) qRT-PCR analysis of linear FAD104 mRNA levels in TNBC cells post-exosome treatment. (D-G) The MTT assay (D), EdU assay (E), wound healing assay (F), Transwell migration and invasion assay (G) of TNBC cells post-exosome treatment. Scale bars, 200 μm. (H) Phalloidin staining of TNBC cell cytoskeleton post-exosome treatment; nuclei stained with DAPI. Scale bars, 50 μm. (I) Western blot analysis of EMT markers in TNBC cells post-exosome treatment; β-actin as loading control. ns, not significant; **P < 0.01; ***P < 0.001.

**Fig. S5 CAF-5-derived exosomal circFAD104 promotes TNBC cell proliferation and stemness.** (A) Table for group serial number labeling. (B) qRT-PCR analysis of circFAD104 levels in TNBC cells post-exosome treatment. (C) qRT-PCR analysis of linear FAD104 mRNA levels in TNBC cells post-exosome treatment. (D) MTT assay, (E) EdU assay (Scale bars, 200 μm), (F) ELDA assay, (G) sphere formation assay (Scale bars, 100 μm) of TNBC cells treated with exosomes from NF-5, CAF-5^si-NC^, or CAF-5^si-circFAD104^. ns, not significant; *P < 0.05; **P < 0.01; ***P < 0.001.

**Fig. S6 CAF-5-derived exosomal circFAD104 enhances TNBC cell metastasis and glycolysis.** (A) Wound healing assay, (B) Transwell migration and invasion assay of TNBC cells post-exosome treatment. Scale bars, 200 μm. (C) Phalloidin staining of TNBC cell cytoskeleton post-exosome treatment; nuclei stained with DAPI. Scale bars, 50 μm. (D) Lactate production, (E) ATP production, (F) glucose consumption levels in TNBC cells treated with CAF5-derived exosomes. (G) Seahorse XF Glycolysis Stress Test measuring extracellular acidification rate (ECAR) in TNBC cells post-CAF5-derived exosome treatment. (H) Western blot analysis of stemness, EMT, and glycolysis markers in TNBC cells post-exosome treatment; β-actin as loading control. *P < 0.05; **P < 0.01; ***P < 0.001.

**Fig. S7 circFAD104 overexpression construct and functional validation.** (A) Schematic of the circFAD104 overexpression plasmid using the pLCDH-ciR vector. (B) qRT-PCR analysis of circFAD104 and linear FAD104 mRNA levels in TNBC cells transfected with empty vector or circFAD104 overexpression plasmid. (C) EdU assay of TNBC cell proliferation post-circFAD104 overexpression. Scale bars, 200 μm. (D) Transwell migration and invasion assay of TNBC cells post-circFAD104 overexpression. Scale bars, 200 μm. (E) Tube formation assay of human umbilical vein endothelial cells (HUVECs) treated with CM from TNBC cells with circFAD104 overexpression. Scale bars, 200 μm. (F) Phalloidin staining of TNBC cell cytoskeleton post-circFAD104 overexpression. Scale bars, 50 μm. (G) Western blot analysis of EMT markers in TNBC cells post-circFAD104 overexpression; β-actin as loading control. ns, not significant; *P < 0.05; **P < 0.01; ***P < 0.001.

**Fig.S8 circFAD104 overexpression regulates cell cycle, apoptosis, stemness, and glycolysis in TNBC cells.** (A) Flow cytometry analysis of cell cycle distribution in TNBC cells post-circFAD104 overexpression. (B) Flow cytometry analysis of apoptosis in TNBC cells post-circFAD104 overexpression (PE Annexin V/7-AAD staining). (C) Sphere formation assay of TNBC cells post-circFAD104 overexpression. Scale bars, 100 μm. (D) Flow cytometry analysis of cancer stem cell (CSC) populations (ALDH⁺ in MDA-MB-231; CD44⁺CD24⁻ in MDA-MB-468) post-circFAD104 overexpression. (E) Immunofluorescence (IF) staining of stemness-related markers (OCT4 and SOX2) in TNBC cells post-circFAD104 overexpression. Nuclei were counterstained with DAPI. Scale bars, 10 μm. (F) Seahorse XF Glycolysis Stress Test measuring ECAR in TNBC cells post-circFAD104 overexpression. (G) Western blot analysis of glycolysis- and stemness- related markers in TNBC cells post-circFAD104 overexpression; β-actin as loading control. (H) Relative glucose consumption, (I) relative lactate production, (J) relative ATP production, (K) intracellular glucose-6-phosphate (G6P), (L) intracellular glucose-1-phosphate (G1P) levels in TNBC cells post-circFAD104 overexpression. ns, not significant; *P < 0.05; **P < 0.01; ***P < 0.001.

**Fig. S9 circFAD104 knockdown suppresses TNBC cell malignant phenotypes.** (A) qRT-PCR validation of circFAD104 knockdown efficiency in TNBC cells; linear FAD104 mRNA levels unaffected. (B) EdU assay of TNBC cell proliferation post-circFAD104 knockdown. Scale bars, 200 μm. (C) Transwell migration and invasion assay of TNBC cells post-circFAD104 knockdown. Scale bars, 200 μm. (D) Tube formation assay of HUVECs treated with CM from TNBC cells with circFAD104 knockdown. Scale bars, 200 μm. (E) Western blot analysis of EMT markers in TNBC cells post-circFAD104 knockdown; β-actin as loading control. ns, not significant; *P < 0.05; **P < 0.01; ***P < 0.001.

**Fig. S10 circFAD104 knockdown regulates cell cycle, apoptosis, stemness, and glycolysis in TNBC cells.** (A) Flow cytometry analysis of cell cycle distribution in TNBC cells post-circFAD104 knockdown. (B) Flow cytometry analysis of apoptosis in TNBC cells post-circFAD104 knockdown (PE Annexin V/7-AAD staining). (C) Sphere formation assay of TNBC cells post-circFAD104 knockdown. Scale bars, 100 μm. (D) Flow cytometry analysis of CSC populations (ALDH⁺ in MDA-MB-231; CD44⁺CD24⁻ in MDA-MB-468) post-circFAD104 knockdown. (E) Immunofluorescence (IF) staining of stemness-related markers (OCT4 and SOX2) in TNBC cells post-circFAD104 knockdown. Nuclei were counterstained with DAPI. Scale bars, 10 μm. (F) Seahorse XF Glycolysis Stress Test measuring ECAR in TNBC cells post-circFAD104 knockdown. (G) Western blot analysis of glycolysis- and stemness-related markers in TNBC cells post-circFAD104 knockdown; β-actin as loading control. (H) Relative glucose consumption, (I) relative lactate production, (J) relative ATP production, (K) intracellular G6P, (L) intracellular G1P levels in TNBC cells post-circFAD104 knockdown. ns, not significant; *P < 0.05; **P < 0.01; ***P < 0.001.

**Fig.S11 2-deoxyglucose (2-DG) reverses circFAD104-mediated pro-tumorigenic phenotypes.** (A) Table for group serial number labeling. (B) qRT-PCR analysis of circFAD104 levels in TNBC cells with circFAD104 overexpression and 2-DG treatment. (C) MTT assay, (D) EdU assay, (E) colony formation assay, and (F) wound healing assay were performed to evaluate the effect of circFAD104 overexpression with or without 2-DG treatment. Scale bars, 200 μm. ns, not significant; **P < 0.01; ***P < 0.001.

**Fig. S12 2-DG reverses CAF-2-derived exosomal circFAD104-mediated proliferation and migration.** (A) qRT-PCR analysis of circFAD104 levels in TNBC cells treated with CAF2^circFAD104^ EXO and 2-DG. (B) MTT assay, (C) EdU assay, (D) Transwell migration assay, (E) wound healing assay of TNBC cells treated with CAF-2^circFAD104^ EXO and 2-DG. Scale bars, 200 μm. ns, not significant; *P < 0.05; ***P < 0.001.

**Fig. S13 2-DG reverses CAF-5-derived exosomal circFAD104-mediated phenotypes and glycolysis.** (A) qRT-PCR analysis of circFAD104 levels in TNBC cells treated with CAF5^circFAD104^ EXO and 2-DG. (B) MTT assay, (C) EdU assay, (D) wound healing assay, (E) Transwell migration assay of TNBC cells treated with CAF-5^circFAD104^ EXO and 2-DG. (F) Relative lactate production levels, (G) relative ATP production levels in TNBC cells treated with CAF-5^circFAD104^ EXO and 2-DG. Scale bars, 200 μm. ns, not significant; *P < 0.05; ***P < 0.001.

**Fig. S14. Identification of circFAD104-interacting candidate proteins and their expression in breast cancer.** (A) Silver staining and Coomassie brilliant blue staining of proteins pulled down by biotin-labeled circFAD104 sense and antisense probes. (B) KEGG pathway enrichment analysis of circFAD104-interacting proteins identified by RNA pull-down followed by mass spectrometry. (C) Pan-cancer analysis of GALM, PDHB, and PGM1 dysregulation across cancer types with breast cancer and its subtypes. (D) Expression analysis of GALM, PDHB, and PGM1 in tumor versus normal tissues across BRCA and its subtypes. *P < 0.05; **P < 0.01; ***P < 0.001.

**Fig. S15 Multi-omic analysis of PDHB and GALM expression and spatial distribution in breast cancer.** (A) Bulk mRNA and protein expression analyses of PDHB in breast cancer using TCGA, GEO and CPTAC datasets. (B) scRNA-seq analysis of PDHB expression across different cell types in multiple BRCA and TNBC datasets. (C) Heatmap showing PDHB expression across cell types in individual BRCA samples. (D) Spatial transcriptomic analysis showing the distribution of PDHB expression in BRCA tissue sections. (E) Bulk mRNA and protein expression analyses of GALM in breast cancer using TCGA, GEO and CPTAC datasets. (F) scRNA-seq analysis of GALM expression across different cell types in multiple BRCA and TNBC datasets. (G) Heatmap showing GALM expression across cell types in individual BRCA samples. (H) Spatial transcriptomic analysis showing the distribution of GALM expression in BRCA tissue sections.

**Fig. S16 Neither PDHB nor GALM is a direct circFAD104-binding partner.** (A-B) qRT-PCR validation of PDHB (A) or GALM (B) knockdown and overexpression efficiencies in TNBC cells. (C-E) Functional analyses of TNBC cells with PDHB knockdown or overexpression, including Transwell migration assay (C), relative intracellular ATP production (D), and relative lactate production (E). Scale bars, 200 μm. (F-H) Functional analyses of TNBC cells with GALM knockdown or overexpression, including Transwell migration assay (F), relative intracellular ATP production (G), and relative lactate production (H). Scale bars, 200 μm. (I-J) RIP assay showing relative enrichment of circFAD104 by Flag-PDHB (I) or Myc-GALM (J). IgG served as the negative control. ns, not significant; *P < 0.05; **P < 0.01; ***P < 0.001.

**Fig. S17. Structural and spatial evidence supporting the circFAD104-PGM1 interaction.** (A) Tandem mass spectrometry (MS/MS) spectrum of PGM1. (B) FISH-IF co-localization assay of circFAD104 and PGM1 in TNBC cells. Scale bar, 20 μm. (C) Secondary structure prediction of circFAD104, including peak profiling and fluctuation analysis.

**Fig. S18 circFAD104 regulates PGM1 protein stability via proteasomal degradation.** (A) qRT-PCR analysis of PGM1 mRNA levels in TNBC cells with circFAD104 overexpression or knockdown. (B) Immunofluorescence staining of PGM1 in TNBC cells with circFAD104 overexpression; nuclei stained with DAPI. Scale bars, 20 μm. (C) Cycloheximide (CHX) chase assay of PGM1 protein half-life in TNBC cells with circFAD104 knockdown. (D) Western blot analysis of PGM1 levels in TNBC cells with MG132 treatment. (E) Western blot analysis of PGM1 levels in TNBC cells with circFAD104 knockdown and MG132/chloroquine (CQ) treatment. ns, not significant; *P < 0.05; **P < 0.01.

**Fig. S19 Multi-omic analysis of PGM1 expression and spatial distribution in breast cancer.** (A) Analysis of PGM1 mRNA levels in breast cancer and adjacent normal tissues using the TCGA-BRCA dataset. (B) Analysis of PGM1 mRNA levels in breast cancer and normal tissues using GSE22820 and GSE62228 datasets. (C) Analysis of PGM1 protein levels in breast cancer and normal tissues using CPTAC data. (D-F) scRNA-seq analysis of PGM1 expression in different cell types from BRCA and TNBC patients. (G) Spatial transcriptomic analysis of PGM1 expression in TNBC tissue sections (tumor nest, tumor-normal junction, stroma). (H-J) Quantitative analysis of PGM1 expression in different spatial regions of TNBC tissues.

**Fig. S20 PGM1 suppresses TNBC cell proliferation in vitro.** (A) qRT-PCR and (B) Western blot validation of PGM1 knockdown and overexpression efficiency in TNBC cells. (C) MTT assay, (D) colony formation assay, (E) EdU assay of TNBC cells post-PGM1 overexpression and knockdown. Scale bars, 200 μm. *P < 0.05; **P < 0.01; ***P < 0.001.

**Fig. S21 PGM1 suppresses TNBC malignant phenotypes in vitro and in vivo.** (A) Wound healing assay (Scale bars, 200 μm), (B) Transwell assay (Scale bars, 200 μm), (C) sphere formation assay (Scale bars, 100 μm) of TNBC cells post-PGM1 knockdown or overexpression. (D) The tumor images, tumor volume, and tumor weight of subcutaneous xenografts derived from MDA-MB-231 cells stably overexpressing PGM1 or vector control. (E) Gross lung images and quantification of pulmonary metastatic nodules in mice intravenously injected with MDA-MB-231 cells stably overexpressing PGM1 or vector control. *P < 0.05; **P < 0.01; ***P < 0.001.

**Fig. S22 PGM1 is associated with glucose metabolism-related pathways in breast cancer.** (A) Enrichment analysis of PGM1-associated genes. (B) Gene set variation analysis (GSVA) of pathway activity in TCGA-BRCA and TNBC datasets (GSE11121, GSE21653) stratified by PGM1 expression.

**Fig. S23 PGM1 regulates glycolytic metabolism in TNBC.** (A) Seahorse XF Glycolysis Stress Test measuring ECAR in TNBC cells with PGM1 knockdown or overexpression. (B-F) Relative glucose consumption (B), lactate production (C), ATP production (D), intracellular G6P (E), and intracellular G1P (F) levels in TNBC cells post-PGM1 knockdown or overexpression. ns, not significant; *P < 0.05; **P < 0.01; ***P < 0.001.

**Fig. S24 PGM1 overexpression reverses circFAD104-mediated pro-tumorigenic phenotypes.** (A) Table for group serial number labeling. (B) Western blot analysis of PGM1 protein levels in TNBC cells with circFAD104 overexpression and/or PGM1 overexpression; β-actin as loading control. (C) MTT assay, (D) colony formation assay, (E) EdU assay, (F) wound healing assay, (G) Transwell assay of TNBC cells with circFAD104 overexpression and/or PGM1 overexpression. Scale bars, 200 μm. ns, not significant; *P < 0.05; **P < 0.01; ***P < 0.001.

**Fig. S25 PGM1 overexpression reverses circFAD104-mediated stemness and glycolytic reprogramming.** (A) Sphere formation assay of TNBC cells with circFAD104 overexpression and/or PGM1 overexpression. Scale bars, 100 μm. (B) Flow cytometry of TNBC cells with circFAD104 overexpression and/or PGM1 overexpression. (C) Relative glucose consumption levels, (D) relative lactate production levels, (E) G6P production levels, (F) G1P production levels in TNBC cells with circFAD104 overexpression and/or PGM1 overexpression. **P < 0.01; ***P < 0.001.

**Fig. S26 PGM1-Δ3+4 truncation mutant reverses circFAD104-mediated malignant phenotypes.** (A) Table for group serial number labeling; Western blot confirmation of PGM1-Δ3+4 expression in TNBC cells; β-actin as loading control. (B) MTT assay, (C) colony formation assay, (D) EdU assay, (E) Transwell migration assay, (F) wound healing assay of TNBC cells with circFAD104 overexpression and/or PGM1-Δ3+4 overexpression. Scale bars, 200 μm. *P < 0.05; **P < 0.01; ***P < 0.001.

**Fig. S27 MARCHF8 interacts with PGM1.** (A) Molecular dynamics simulation of MARCHF8 and PGM1. (B) Endogenous co-immunoprecipitation (co-IP) assay confirming MARCHF8-PGM1 interaction in TNBC cells. (C) Immunofluorescence assay of the co-localization between MARCHF8 and PGM1 in TNBC cells; nuclei stained with DAPI. Scale bars, 10 μm. (D) Schematic of Myc-tagged MARCHF8 truncation mutants. (E) qRT-PCR analysis of MARCHF8 mRNA levels in TNBC cells with MARCHF8 overexpression or knockdown; β-actin as internal control. ***P < 0.001.

**Fig. S28 circFAD104 regulates MARCHF8-PGM1 interaction.** (A) qRT-PCR analysis of MARCHF8 mRNA levels in TNBC cells with circFAD104 knockdown or overexpression. (B) Western blot analysis of MARCHF8 protein levels in TNBC cells with circFAD104 knockdown or overexpression; β-actin as loading control. (C) Immunofluorescence staining of MARCHF8 subcellular localization in TNBC cells with circFAD104 overexpression; nuclei stained with DAPI. Scale bars, 10 μm. (D) FISH-IF co-localization assay of circFAD104 and MARCHF8 in TNBC cells with circFAD104 overexpression. Scale bars, 20 μm. (E) Endogenous co-IP assay of MARCHF8-PGM1 interaction in TNBC cells with circFAD104 overexpression. (F) Endogenous co-IP assay of MARCHF8-PGM1 interaction in TNBC cells with circFAD104 knockdown. ns, not significant.

**Fig. S29 circFAD104 overexpression in TNBC cells promotes tumor growth and metastasis in vivo.** (A) The tumor images, tumor weight, and tumor volume measurements in nude mice implanted with MDA-MB-231 cells stably overexpressing circFAD104 or the empty vector. Scale bars, 1 cm. (B) Relative lactate and ATP production levels in xenograft tissues. (C) qRT-PCR analysis of circFAD104 expression in xenograft tissues. (D) Representative HE, ISH analysis of circFAD104, IHC staining of related markers in xenograft tissues. Scale bars, 100 μm. (E) Western blot analysis of EMT, stemness, and glycolysis markers in xenograft tissues; β-actin as loading control. (F) In vivo bioluminescent imaging and gross images of lungs from mice injected with MDA-MB-231 cells stably overexpressing circFAD104 or the empty vector. (G) Quantitative analysis of pulmonary metastatic nodules. (H) HE staining of lung tissues showing metastatic foci. Scale bars, 100 μm. *P < 0.05; **P < 0.01; ***P < 0.001.

**Fig. S30 circFAD104 expression and clinical relevance in the discovery and validation cohorts**. (A) The clinicopathological features of breast cancer patients in the discovery cohort (n = 54). (B) The clinicopathological features of breast cancer patients in the independent validation cohort (n = 95). (C) Kaplan-Meier survival analysis and ROC curves evaluating the prognostic performance of circFAD104 expression in CAF-enriched stromal and tumor-cell compartments of TNBC tissues in the validation cohort.

**Table S1.** **The clinicopathological features of breast cancer patients in the discovery cohort .**

| **Variables** | **Cases (%)** | **CircFAD104**  **expression in CAFs** | | **P value** | **CircFAD104**  **expression in BC cells** | | **P value** |
| --- | --- | --- | --- | --- | --- | --- | --- |
|  |  | **Low**  **n=26** | **High**  **n=28** |  | **Low**  **n=27** | **High**  **n=27** |  |
| **Age** |  |  |  |  |  |  |  |
| <50 | 27(50.0%) | 15 | 12 | 0. 276a | 14 | 13 | 0.786a |
| ≥50 | 27(50.0%) | 11 | 16 |  | 13 | 14 |  |
| **Tumor size** |  |  |  |  |  |  |  |
| <2cm | 19(35.1%) | 9 | 10 | 0.933a | 7 | 12 | 0.154a |
| ≥2cm | 35(64.8%) | 17 | 18 |  | 20 | 15 |  |
| **Lymphatic**  **metastasis** |  |  |  |  |  |  |  |
| Negative | 30(55.5%) | 18 | 12 | 0.051a | 17 | 13 | 0.273a |
| Positive | 24(44.4%) | 8 | 16 |  | 10 | 14 |  |
| **Distant metastasis** |  |  |  |  |  |  |  |
| Negative | 42(77.7%) | 24 | 18 | **0.013b** | 25 | 17 | **0.009b** |
| Positive | 12(22.2%) | 2 | 10 |  | 2 | 10 |  |
| **Pathological grade** |  |  |  |  |  |  |  |
| G1+G2 | 22(40.8%) | 12 | 10 | 0.435a | 12 | 10 | 0.580a |
| G3  **Ki67** | 32(59.2%) | 14 | 18 |  | 15 | 17 |  |
| ≤20% | 7(13.0%) | 2 | 5 | 0.267b | 2 | 5 | 0.224b |
| >20% | 47(87.0%) | 24 | 23 |  | 25 | 22 |  |

a Pearson's chi-squared test

b Fisher's exact test

**Table S2. The clinicopathological features of breast cancer patients in the validation cohort.**

| **Variables** | **Cases**  **(%)** | **CircFAD104 expression in CAFs** | | **P value** | **CircFAD104 expression in BC cells** | | **P value** |
| --- | --- | --- | --- | --- | --- | --- | --- |
|  |  | **Low**  **n=52** | **High**  **n=43** |  | **Low**  **n=58** | **High**  **n=37** |  |
| **Age** |  |  |  |  |  |  |  |
| <50 | 38(40.0%) | 22 | 16 | 0.614a | 24 | 14 | 0.731a |
| ≥50 | 57(60.0%) | 30 | 27 |  | 34 | 23 |  |
| **Tumor size** |  |  |  |  |  |  |  |
| <2cm | 29(30.5%) | 16 | 13 | 0.955a | 18 | 11 | 0.893a |
| ≥2cm | 66(69.4%) | 36 | 30 |  | 40 | 26 |  |
| **Lymphatic**  **metastasis** |  |  |  |  |  |  |  |
| Negative | 51(53.6%) | 35 | 18 | **0.0129a** | 38 | 15 | **0.0168a** |
| Positive | 44(46.3%) | 17 | 25 |  | 20 | 22 |  |
| **Distant**  **metastasis** |  |  |  |  |  |  |  |
| Negative | 71(74.7%) | 45 | 26 | **0.00360a** | 50 | 21 | **0.00128a** |
| Positive | 24(25.2%) | 7 | 17 |  | 8 | 16 |  |
| **Pathological grade** |  |  |  |  |  |  |  |
| G1+G2 | 52(54.8%) | 29 | 23 | 0.824a | 29 | 23 | 0.246a |
| G3 | 43(45.2%) | 23 | 20 |  | 29 | 14 |  |
| **Ki67** |  |  |  |  |  |  |  |
| ≤20% | 17(17.8%) | 9 | 8 | 0.870a | 9 | 8 | 0.449a |
| >20% | 78(82.1%) | 43 | 35 |  | 49 | 29 |  |

a Pearson's chi-squared test

**Table S3. Antibodies used in the experiments**

| **Antigen** | | **Supplier** | **Catalog#** | **Application** |
| --- | --- | --- | --- | --- |
| AKT | | Beyotime | AF0045 | IB(1:1000) |
| ALDOA | | Proteintech | 11217-1-AP | IB(1:5000) |
| Calnexin | | Cell Signaling Technology | 2679 | IB (1:1000) |
| CD9 | | Cell Signaling Technology | 98327 | IB (1:1000) |
| CD63 | | Cell Signaling Technology | 52090 | IB (1:1000) |
| E-Cadherin | | Selleck | F0005 | IB(1:1000) IHC(1:100) |
| Fibronectin | | ABCAM | ab6328 | IB(1:1000) |
| FAP | | Cell Signaling Technology | 66562 | IB (1:1000) IF(1:200) |
| FSP1 | | Proteintech | 16105-1-AP | IB (1:1000) IF(1:200) |
| FLAG TAG | | Invitrogen | PA1-984B | IB(1:1000) |
| GLUT1 | | Selleck | F1192 | IB(1:1000) |
| GM130 | | Cell Signaling Technology | 12480 | IB (1:1000) |
| HSP70 | | Cell Signaling Technology | 4872 | IB (1:1000) |
| HA TAG | | Thermo Fisher | PA1-985 | IB(1:1000) |
| HRP-anti-mouse | | Cell Signaling Technology | 7076 | IB (1:5000) |
| HRP-anti-rabbit | | Cell Signaling Technology | 7074 | IB (1:5000) |
| HK1 | | Proteintech | 15656-1-AP | IB(1:1000) IHC(1:100) |
| HK2 | | Proteintech | 22029-1-AP | IB(1:1000) IHC(1:100) |
| Ki67 | | Proteintech | 10197-1-AP | IHC(1:100) |
| LDHA | | Selleck | F0233 | IB(1:1000) IHC(1:100) |
| MYC TAG | | Bethyl | A190-105A | IB(1:1000) |
| MARCHF8 | | Proteintech | 14119-1-AP | IB (1:1000) IF(1:200) |
| NANOG | Proteintech | | 67255-1-Ig | IB(1:1000) IHC(1:100) |
| N-cadherin | | Affinity | af5239 | IB(1:1000) IHC(1:100) |
| NF-κB | | Proteintech | 66535-1-Ig | IB(1:1000) |
| OCT4 | | Proteintech | 11263-1-AP | IB(1:1000) IHC(1:100) |
| p-AKT | | Proteintech | 66444-1-Ig | IB(1:1000) |
| PGM1 | | Proteintech | 66105-1-Ig | IB (1:1000) IF(1:200)  IHC(1:100) |
| PFKP | | Proteintech | 13389-1-AP | IB(1:1000) |
| p-Ikβ-α | | Cell Signaling Technology | 2859T | IB(1:1000) |
| p-STAT3 | | Affinity | AF3293 | IB(1:1000) |
| SLUG | | Selleck | F0362 | IB(1:1000) IHC(1:100) |
| SOX2 | | Beyotime | AF8034 | IB(1:1000) IHC(1:100) |
| β-Actin | | Proteintech | 66009-1-Ig | IB(1:1000) |
| β-CATENIN | | Beyotime | AF1189 | IB(1:1000) |
| α-SMA | | Cell Signaling Technology | 19245 | IB (1:1000) IF(1:200)  IHC(1:400) |
| Vimentin | | Cell Signaling Technology | 5741 | IB (1:1000) IF(1:200) |

**Table S4. siRNAs used for transfection**

| **Name** | **sense （5’-3’）** | **antisense （5’-3’）** |
| --- | --- | --- |
| si-circFAD104 | AGAUUAUCAUGUGAGAAAU | AUUUCUCACAUGAUAAUCU |
| si-PGM1 | GUGGGACCGUAUGUAAAGA | UCUUUACAUACGGUCCCAC |
| si-PDHB | CCAUUGACCAGGUUAUAAA | UUUAUAACCUGGUCAAUGG |
| si-GALM | GAGAGCUCAUAGUCAACUA | UAGUUGACUAUGAGCUCUC |
| si-MARCHF8 | GGACUAAAUUGGUGGUUGU | ACAACCACCAAUUUAGUCC |
| si-NC | UUCUCCGAACGUGUCACGU | ACGUGACACGUUCGGAGAA |

**Table S5. Primers used for qRT-PCR**

| **Gene** | **Forward （5’-3’）** | **Reverse （5’-3’）** |
| --- | --- | --- |
| circFAD104 | GCTCCCCCTGTTGGACTTTC | GAGGCTTGCTGTACTGGTCTT |
| FAD104 | CCATTCATGTGCCTCCAG | GGTGAGTCAGATAGGGAGG |
| PGM1 | TGATGGACGCGAGCAAACTGTC | ATGTCCTCCACACTCTGCTTGC |
| PDHB | TGTAACTGTGGAAGGAGGCTGG | CATCAGCACCAGTGACACGAAC |
| GALM | CAACCGAATCGCCAAAGGAACC | CGAGAACTGGACGCCATTTGAC |
| MARCHF8 | CTCTCGCACTTCTATCACGCCA | AAGTGGAGGCTTCCTGTGCAGT |
| U6 | CTCGCTTCGGCAGCACA | AACGCTTCACGAATTTGCGT |
| GAPDH | GGAGCGAGATCCCTCCAAAAT | GGCTGTTGTCATACTTCTCATGG |
| Actin | CACTGTGCCCATCTACGAG | AATGTCACGCACGATTTCC |
